# Potential Harms of Feedback After Web-Based Depression Screening: Secondary Analysis of Negative Effects in the Randomized Controlled DISCOVER Trial

**DOI:** 10.2196/59476

**Published:** 2025-04-30

**Authors:** Franziska Sikorski, Bernd Löwe, Anne Daubmann, Sebastian Kohlmann

**Affiliations:** 1 Department of Psychosomatic Medicine and Psychotherapy University Medical Center Hamburg-Eppendorf Hamburg Germany; 2 Department of Medical Biometry and Epidemiology University Medical Center Hamburg-Eppendorf Hamburg Germany; 3 Department of General Internal Medicine and Psychosomatics University Medical Centre Heidelberg Heidelberg Germany

**Keywords:** randomized controlled trial, depressive disorder, screening, negative effects, harms, web-based, mental health, misdiagnosis, overdiagnosis

## Abstract

**Background:**

Web-based depression screening followed by automated feedback of results is frequently used and promoted by mental health care providers. However, criticism points to potential associated harms. Systematic empirical evidence on postulated negative effects is missing.

**Objective:**

We aimed to examine whether automated feedback after web-based depression screening is associated with misdiagnosis, mistreatment, deterioration in depression severity, deterioration in emotional response to symptoms, and deterioration in suicidal ideation at 1 and 6 months after screening.

**Methods:**

This is a secondary analysis of the German-wide, web-based, randomized controlled DISCOVER trial. Affected but undiagnosed individuals screening positive for depression (9-item Patient Health Questionnaire [PHQ-9] ≥10 points) were randomized 1:1:1 to receive nontailored feedback, tailored feedback, or no feedback on their screening result. Misdiagnosis and mistreatment were operationalized as having received a depression diagnosis by a health professional and as having started guideline-based depression treatment since screening (self-report), respectively, while not having met the *Diagnostic and Statistical Manual of Mental Disorders* (Fifth Edition) (*DSM-V*) criteria of a major depressive disorder at baseline (Structured Clinical Interview for *DSM-V* Disorders). Deterioration in depression severity was defined as a pre-post change of ≥4.4 points in the PHQ-9, deterioration in emotional response to symptoms as a pre-post change of ≥3.1 points in a composite scale of the Brief Illness Perception Questionnaire, and deterioration in suicidal ideation as a pre-post change of ≥1 point in the PHQ-9 suicide item. Outcome rates were compared between each feedback arm and the no feedback arm in terms of relative risks (RRs).

**Results:**

In the per protocol sample of 948 participants (n=685, 72% female; mean age of 37.3, SD 14.1 years), there was no difference in rates of misdiagnosis (ranging from 3.5% to 4.9% across all study arms), mistreatment (7.2%-8.3%), deterioration in depression severity (2%-6.8%), deterioration in emotional response (0.7%-2.9%), and deterioration in suicidal ideation at 6 months (6.8%-13.1%) between the feedback arms and the no feedback arm (RRs ranging from 0.46 to 1.96; *P* values ≥.13). The rate for deterioration in suicidal ideation at 1 month was increased in the nontailored feedback arm (RR 1.92; *P*=.01) but not in the tailored feedback arm (RR 1.26; *P=*.43), with rates of 12.3%, 8.1%, and 6.4% in the nontailored, tailored, and no feedback arms, respectively. All but 1 sensitivity analyses as well as subgroup analyses for false-positive screens supported the findings.

**Conclusions:**

The results indicate that feedback after web-based depression screening is not associated with negative effects such as misdiagnosis, mistreatment, and deterioration in depression severity or in emotional response to symptoms. However, it cannot be ruled out that nontailored feedback may increase the risk of deterioration in suicidal ideation. Robust prospective research on negative effects and particularly suicidal ideation is needed and should inform current practice.

**Trial Registration:**

ClinicalTrials.gov NCT04633096; https://clinicaltrials.gov/study/NCT04633096; Open Science Framework 10.17605/OSF.IO/TZYRD; https://osf.io/tzyrd

## Introduction

### Background

Depressive disorders, although being among the most disabling and most prevalent disorders worldwide [1], often remain undetected and therefore untreated [2]. In the last decades, depression screening has been increasingly discussed as promising to reach those affected but undetected at an early stage. In addition to population-level screening in routine clinical care, as, for example, recommended in the United States [3], advocates also speak out in favor of screening for depression on the web [4]. For many affected individuals, the web is already the favored source for information on mental health [5,6]. Furthermore, so-called web-based depression tests are widely promoted by mental health–related institutions and frequently used by those seeking diagnostic advice [7]. The rationale of web-based depression tests typically involves administering symptom-based screening questionnaires and providing individuals with direct feedback on screening results, sometimes supplemented by direct links or referrals to services. The feedback is thought to empower affected individuals to better act on their symptoms [8] and to seek diagnostic consultation and, if necessary, appropriate care. As such, it might improve early detection and management of depression.

However, feedback after web-based depression screening has been proposed and implemented without due consideration of its appropriateness, that is, without evaluating its effectiveness against the background of potential negative effects. While assessing negative effects is recommended for all clinical interventions, it is particularly important in screening interventions, as in these per definition a substantial amount of participants will not benefit [9]. In addition, in web-based contexts there is no experienced health care stuff available to monitor participants who might need support [10]. As such, the balance between harms and benefits of feedback after web-based depression screening could easily lean toward harms. Indeed, there is no empirical evidence of positive effects on targeted patient-related outcomes: 2 randomized controlled trials, 1 by Batterham and colleagues [11] and the DISCOVER trial recently conducted by our research team [12], do not indicate that feedback of depression-screening results promotes the uptake of evidence-based depression care or reduces depression severity. Negative effects, if present, would therefore likely be generated without creating substantial health benefits.

Evidence regarding negative effects, however, is scarce, with the current scientific debate being mainly reflected by opinion papers. The first area of negative effects of depression screening, discussed in both medical and web-based contexts, relates to inadequate management and care for individuals who receive false-positive feedback. Critics particularly point to the risk of increased rates of misdiagnosis and mistreatment, which refers to the allocation of depression diagnosis and treatment by health care professionals to individuals who screen positive but do not meet diagnostic criteria for a depressive disorder. This, again, is assumed to lead to unnecessary iatrogenic effects such as adverse medication and psychotherapy side effects in healthy individuals, societal costs, and waste of limited health care resources resulting in potential undertreatment of more severe cases [4,13,14]. A second area of concern relates to negative psychological effects to the feedback of screening results. In the field of breast cancer screening, for example, it is well established that screening and particularly false-positive results may be associated with increased anxiety and distress [15]. With regard to depression-screening feedback, it is similarly assumed that feedback-induced labeling, resembling a clinical diagnosis, might induce anxiety, distress, stigma, or nocebo effects as, for example, deterioration of symptoms [4,14,16]. These effects could be amplified by the fact that, in contrast to medical settings, in web-based depression screening, the “diagnosis” would be delivered without a health professional who could provide emotional support or advice on further steps [17]. Indeed, in qualitative studies on web-based mental health screening, some participants describe having been discouraged, shocked, or concerned by the feedback they received [8,18]. Furthermore, 1 observational study found that screening procedures including referrals to in-person care had a higher likelihood of subsequent web-based searches for suicidal intent, potentially suggesting a deterioration of suicidal ideation [7]. In contrast, in our recently conducted DISCOVER trial on feedback after web-based depression screening, less than 1% of participants qualitatively reported any negative effect attributed to trial participation when asked via telephone 6 months after screening, with no indication for an association of negative effects with the recommendation to seek diagnostic advice (see the study by Kohlmann et al [12] for previously published results). However, systematic and large-scale quantitative research on the discussed potential negative effects is outstanding.

### Objectives

In this study, we addressed this lack of evidence by analyzing data from our recently conducted randomized controlled DISCOVER trial on feedback after web-based depression screening [12]. In extension to efficacy findings and qualitative reports on negative effects published previously [12], this secondary analysis quantitatively evaluates the potential negative effects discussed in the literature outlined previously (regarding misdiagnosis, mistreatment, and psychological negative effects). Specifically, we aimed to examine whether feedback after web-based depression screening is associated with increased misdiagnosis and mistreatment 6 months after screening, as well as deterioration in depression severity, deterioration in emotional response to symptoms, and deterioration in suicidal ideation 1 and 6 months after screening.

## Methods

### Study Design and Participants

The DISCOVER trial [19] and this secondary analysis [20] were preregistered. We conducted small deviations from the preregistration: we added the outcomes misdiagnosis and emotional response to symptoms, as we deemed this of clinical interest. Furthermore, we added sensitivity analyses based on logistic regression models. The detailed study protocol [18] and main results of the trial [12] have been described previously. Data collection was conducted on the web and in the German language between January 12, 2021, and September 30, 2022; this secondary data analysis was conducted between May 3, 2023, and December 23, 2023.

DISCOVER was an investigator-initiated, observer-blinded, 3-armed, randomized controlled trial that compared automated feedback with no feedback after web-based depression screening. After being screened for depression with the digitized 9-item Patient Health Questionnaire (PHQ-9 [21]), eligible participants were randomized to receive no feedback, nontailored feedback, or tailored feedback on their screening result (1:1:1 allocation ratio). Assessments were set at baseline and 1- and 6-month follow-ups. In this secondary analysis, we compared rates of misdiagnosis and mistreatment (at 6 months) as well as deterioration in depression severity, deterioration in emotional response to symptoms, and deterioration in suicidal ideation (at 1 and 6 months) between each feedback arm and the no feedback arm.

Participants were 18 years or older with at least moderate depression severity (PHQ-9 ≥10) but not diagnosed with or treated for depression within the last year. Additional eligibility criteria were having sufficient web-based literacy and German language proficiency, providing contact details, and giving web-based informed consent.

### Study Procedures

The study was promoted as being on “stress and psychological well-being” on a publicly accessible study website [22] from January 2021 to January 2022. The aim of evaluating web-based depression screening was not explicitly communicated, but interested participants were informed that some of them will get feedback on a part of their answers. Traditional and social media campaigns as well as print advertisements in public areas of several German cities were used to approach interested individuals across Germany. To reach a sample that strives for representativeness of the German population with respect to age and gender, a marketing company further advertised the study via a nationwide web-based access survey panel.

After completing baseline assessment and screening, eligible participants were automatically randomized by random permuted blocks randomization stratified for baseline depression severity (moderate: PHQ-9 score 10-14 points; severe: PHQ-9 score ≥15 points) and allocated 1:1:1 to 1 of the 3 study arms. Double entries identified based on personal data by a privacy-preserving record linkage service [23] were automatically reallocated to their former study arm. Research staff were masked to allocation at any time until breaking the blind. Due to the design, participants could not be masked but were kept unaware of trial hypotheses to minimize expectancy bias.

Web-based follow-up assessments were set at 1 month and 6 months after randomization, with up to 10 automatic email reminders being sent to participants in case of incomplete surveys. Two to five days and 6 months after randomization, participants were contacted via telephone for complementary diagnostic interviews, with calls being repeated at different hours during daytime and evening in case participants were not reached (see the study protocol by Sikorski et al [18] for more detailed information on data collection procedures).

### Web-Based Depression Screening and Feedback of Screening Results

Participants underwent depression screening as part of the baseline survey using the digitized PHQ-9 [21,24] (see Multimedia Appendix 1 for the layout of the digitized version). The use of the PHQ-9 for web-based depression screening is justified by several reasons. First, at the standard cutoff value of ≥10 points, the paper-pencil PHQ-9 demonstrates high discriminatory performance for detecting a major depressive disorder. Based on a recent individual participant data meta-analysis of studies with a semistructured interview reference standard, pooled PHQ-9 sensitivity and specificity (95% CI) were 0.85 (0.79-0.89) and 0.85 (0.82-0.87), respectively [25]. Second, preliminary evidence suggests that psychometric characteristics for the PHQ-9 are comparable for the digitized version [26,27]. Third, the PHQ-9 is recommended for depression screening by the US Preventive Services Task Force and the German National Clinical Practice Guideline for Unipolar Depression [3,28].

**Figure 1:**
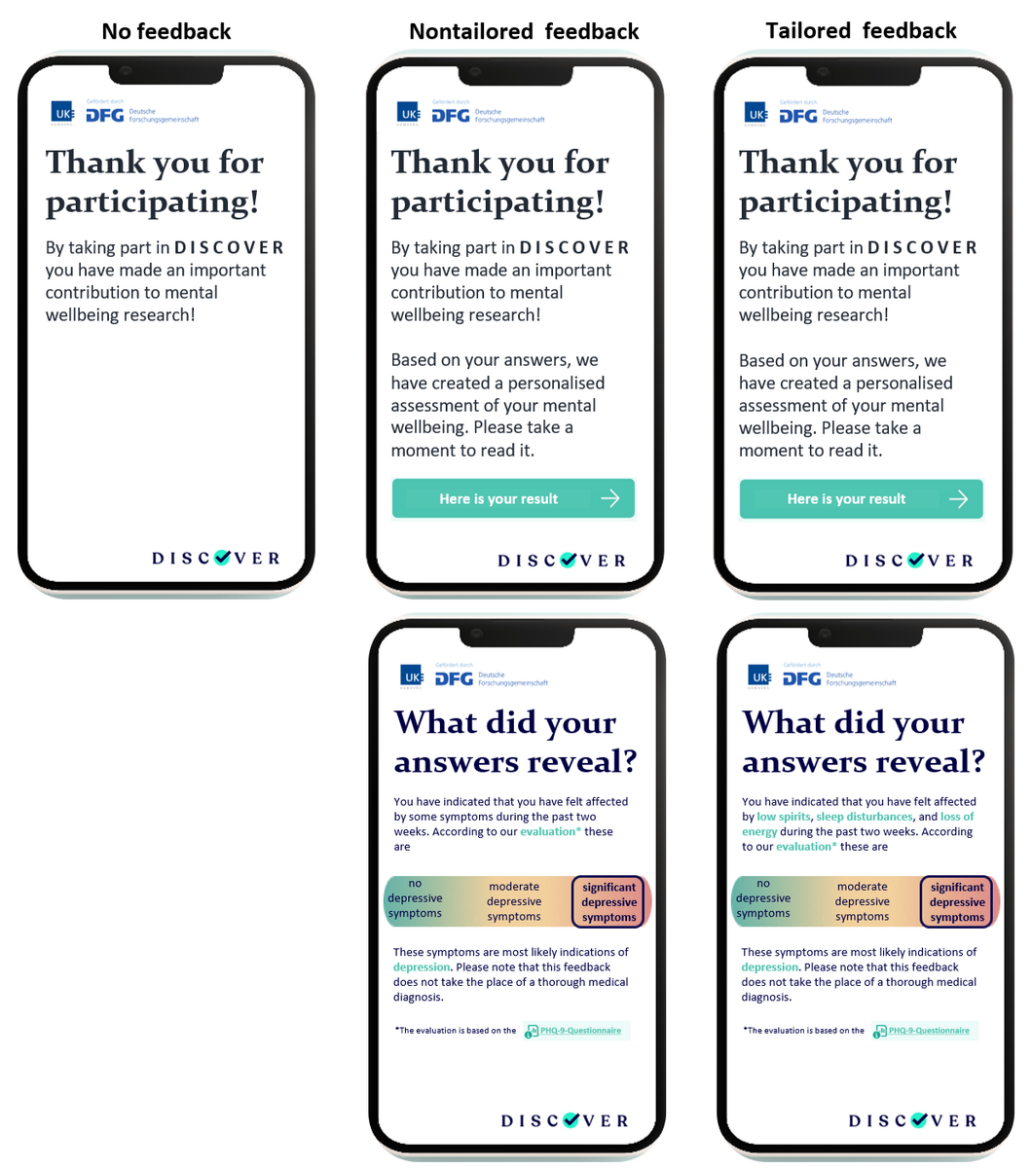
Illustrations of no feedback, nontailored feedback (first screen), and tailored feedback (first screen; reprinted from the study by Kohlmann et al [12]).

After completing the baseline survey, all participants were thanked for participating in the study and received information on follow-up procedures. Participants of the feedback arms were additionally offered feedback on their screening result by clicking on a “next” button (Figure 1 [12]). Both nontailored and tailored feedback comprised four sections: (1) the depression screening result indicating the presence of “significant depressive symptoms,” (2) a note to seek diagnostic consultation by a health professional together with a link to make an appointment within the next 2 weeks, (3) brief general information on depression, and (4) information on depression treatment based on the German National Clinical Practice Guideline for Unipolar Depression [28]. Notably, in the German health care system depression care is available and covered by the social health insurance. Information was extended by direct links to referenced health or social services (eg, web-based therapies covered by the health insurance and self-help groups), and the feedback form could be downloaded in a file that included all hyperlinks. In extension to the nontailored feedback, the information in the tailored feedback intervention was personalized to participants’ characteristics (eg, “You have indicated that you had l*ow spirits, sleep disturbances,* and *loss of energy* during the past two weeks.”). In addition, after being provided with the screening result (section 1) but before receiving further information (sections 2-4), participants were asked whether they think that their symptoms were indications of depression and whether they worried about the symptoms. According to the participants’ answers, the following 3 feedback sections were arranged in a differing order, phrased slightly differently, and extended by information tailored to participants’ risk profile (eg, “Depression *in pregnancy* is common.”). The feedback was developed in a multistage process involving patient representatives [29,30] and a digital graphic agency to adapt the material to the possibilities of web-based presentation. Illustrations of the complete nontailored and tailored feedback versions can be found in Multimedia Appendices 2 and 3.

Due to ethical considerations, all participants who have indicated elevated suicidal ideation (PHQ-9 suicide item ≥2; *more than half the days*) were shown a screen providing advice to urgently seek help and relevant information on available help services (eg, general practitioner, local psychiatric emergency units, and the national emergency number; Multimedia Appendix 4).

### Measures

Depression diagnosis by a health professional was assessed at 6 months with the question: “Have you been diagnosed with depression or burnout in the last six months?”

Guideline-based depression treatment, that is, pharmacotherapy with antidepressant medication or psychotherapy recommended by the German National Clinical Practice Guideline for Unipolar Depression [28], was assessed at 6 months with the questions: “Have you started any psychotherapy or similar treatment in the last 6 months [which]?”) and “Have you started taking medication to treat depression or other complaints such as sleep problems, anxiety or stress [which ones]?” Participants could choose from guideline-based treatment options or give open answers. In case of open answers, these were checked for guideline conformity independently by 2 of the authors (SK and FS).

Criteria for a major depressive disorder at baseline were assessed with the depression-related modules of the Structured Clinical Interview for *DSM-V* Disorders (SCID-5-CV) [31] 2-5 days after screening. The interviewers (MSc psychology students) were trained and supervised by the project leader, who is an experienced psychotherapist. Participants who did not meet the criteria for a major depressive disorder according to the SCID were considered false-positive screens.

Depression severity was assessed with the PHQ-9 at 1 and 6 months after screening. In accordance with the *Diagnostic and Statistical Manual of Mental Disorders* (Fifth Edition) (*DSM-V*) diagnostic criteria, the PHQ-9 assesses 9 depressive symptoms each rated in terms of frequency during the past 2 weeks (0-3; *not at all* to *nearly every day*), resulting in a total score ranging from 0 to 27, with a higher score indicating higher depression severity. The PHQ-9 is among the most frequently used self-report depression questionnaires, has good psychometric properties, and is sensitive to change [21,32].

Suicidal ideation was assessed with the PHQ-9 suicide item (item 9): “Over the last two weeks, how often have you been bothered by thoughts that you would be better off dead or of hurting yourself in some way?” rated from 0 to 3 (*not at all, several days, more than half the days,* and *nearly every day*).

Emotional response to depressive symptoms was assessed with a composite scale based on 2 items of the Brief Illness Perception Questionnaire (Brief IPQ) that cover emotional representations of depressive symptoms: “How concerned are you about your symptoms?” and “How much do your symptoms affect you emotionally? (eg, do they make you angry, scared, upset or depressed)?” The items were assessed directly after the PHQ-9 and were scored on a Likert scale ranging from 0 (*not at all*) to 10 (*extremely*). Item scores were pooled for the composite scale, resulting in a total scale ranging from 0 to 10. The respective items of the Brief IPQ showed good psychometric properties [33].

### Outcomes

Participants were classified as misdiagnosed or mistreated if they reported having received a depression diagnosis by a health professional or guideline-based depression treatment while not having met the criteria for a major depressive disorder at baseline (SCID), that is, while being screened false positive.

Deterioration in depression severity was defined as a pre-post change score of at least 4.4 points in the PHQ-9. The cutoff is based on the reliable change index (RCI), a psychometric criterion to evaluate whether a change in symptoms is considered statistically reliable, that is, not attributable to measurement error [34]. The RCI was calculated using the PHQ-9 SD from the current sample (SD_baseline_=4), the reliability coefficient from the PHQ-9 validation study (*r*_tt_=0.84) [21], and a 95% CI. The resulting RCI of 4.4 points is comparable with cutoffs found in comparable research [32,35].

Deterioration in emotional response to depressive symptoms was defined as a pre-post change score of at least 3.1 points in the relating composite scale. The RCI was calculated using the SD of this composite scale (SD_baseline_=1.9), the pooled reliability coefficients from the Brief IPQ validation study (*r*_tt_=0.66), and a 95% CI.

Deterioration in suicidal ideation was defined as the pre-post change score of at least 1 point in the PHQ-9 suicide item.

### Sample

We performed this secondary analysis in the per protocol sample that included 89% (948/1178) of randomized participants who had at least one postbaseline value of one of the outcomes and no major protocol violation. Major protocol deviations were predefined as not receiving or adhering to the intervention (ie, feedback not opened, feedback reading time 15 seconds, or no download of feedback form), multiple participation (post hoc data check or self-report), reports of not having answered the survey seriously, baseline survey completion time less than 2 minutes, and provision of an invalid email address. We preferred per protocol over intention-to-treat (ITT) analysis, as the second is likely to underestimate the risk of an event by inflating the denominator with participants who have provided invalid data or have never received the intervention. Whereas this is conservative in efficacy evaluations, in the current case of a risk evaluation we consider it more appropriate to prevent failing to detect a risk than overestimating it [36].

In addition, we performed sensitivity analyses in ITT sample, both with and without missing data imputation. We used 2 strategies for imputing data: assuming that all dropouts were deteriorators, considering this to be the most conservative estimate (worst case); and assuming that all dropouts were nondeteriorators, considering this to be the most optimistic estimate (best case).

### Statistical Analyses

We compared the rates of negative effects between study arms in terms of relative risks (RRs). The RR estimates how much higher (or lower) the probability of negative effects is for participants in each feedback arm compared with the no feedback arm. To directly estimate the RR with 95% CIs, we applied generalized linear models with a log link and robust sandwich variance estimator using modified log-Poisson regressions [37]. We chose this approach over alternative models as it is suited as well in case of frequent outcomes and suffers least from convergence problems [38,39]. To test for differential effects in the subgroup of false-positive screened participants, we ran another series of models additionally including false-positive screens and the false-positive screen by study arm interaction term. We set the significance level at α=.05 and did not correct for multiple testing for 2 reasons: the trial was not powered for this secondary analysis, and as already mentioned, in case of negative effects we consider it more important to prevent the inflation of a type II error (ie, failing to detect negative effects in case they exist) instead of the type I error. As some negative effects turned out to be rare in the study data, we also estimated odds ratios based on logistic regression models as post hoc sensitivity analyses. We performed analyses with SPSS software (version 27; IBM Corp).

### Ethical Considerations

The data used in this secondary analysis are derived from the DISCOVER RCT, which has been reviewed and approved by the ethics committee of the Hamburg Medical Chamber (# PV7039). The study and reporting of this manuscript followed appropriate CONSORT (Consolidated Standards of Reporting Trials) guidelines, including the harms and the eHealth statement [9,40-44] (Multimedia Appendix 5). Participants received detailed study information, including information on their ability to withdraw from the study at any time and without giving reasons. Web-based informed consent, covering the use of data for secondary analyses, was obtained from all participants via checkboxes. Participation was compensated with Amazon vouchers worth up to €15 (US $17.11; one €5, US $5.70, voucher per complete follow-up assessment). The data were deidentified (pseudonymized) after the completion of data collection.

## Results

### Overview

**Figure 2:**
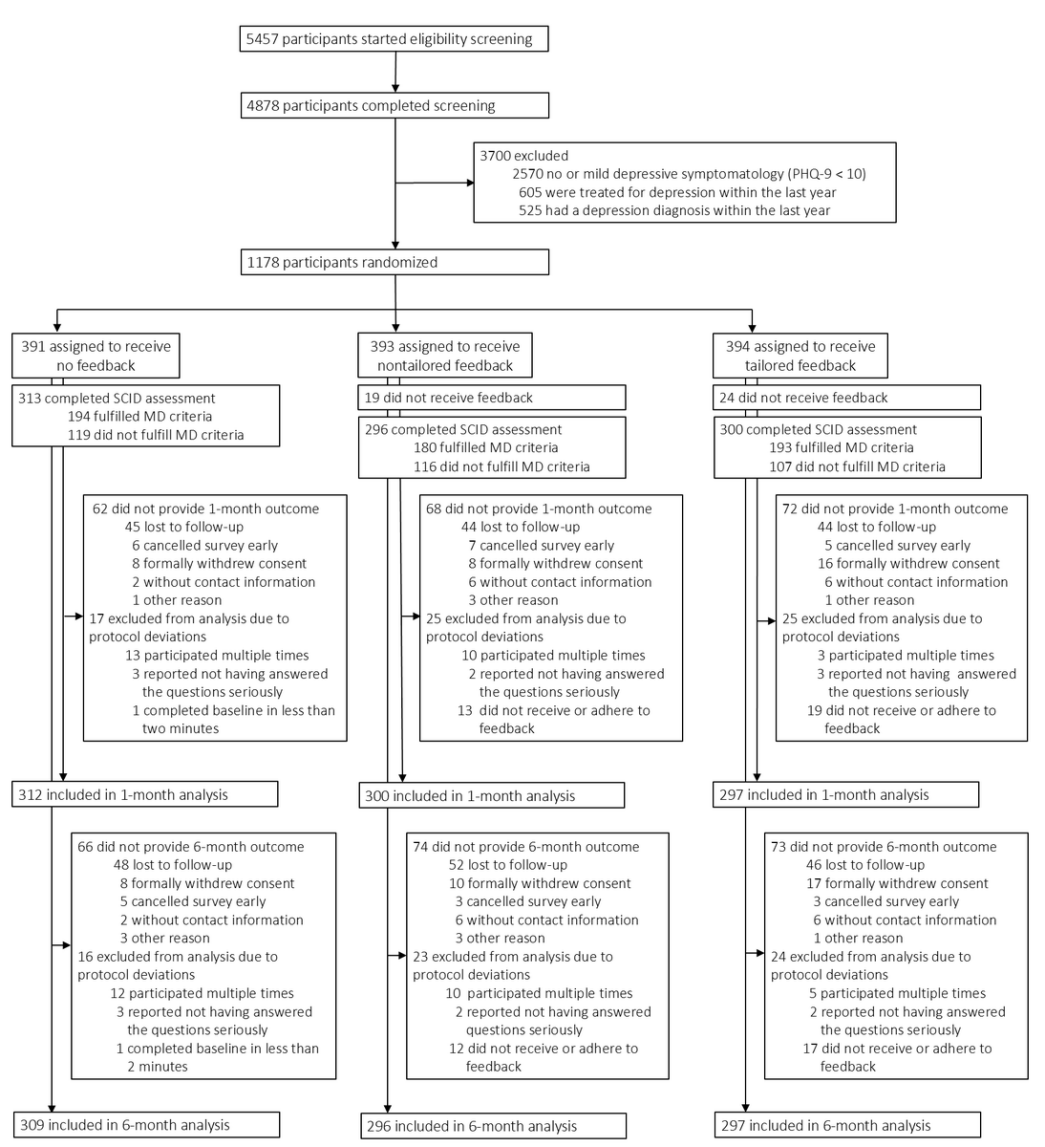
CONSORT flowchart (per protocol sample). PHQ-9: 9-item Patient Health Questionnaire-9; SCID: Structured Clinical Interview for DSM-V Disorders.

Of initially 5457 study participants, 4878 completed the screening questionnaire, and 1178 eligible participants were assigned to receive no feedback (n=391), nontailored feedback (n=393), or tailored feedback (n=394) on their depression screening result. Of the 787 participants randomized to receive any feedback, 95% (n=744) opened the feedback screen, of whom 62% (n=464) downloaded the PDF and 33% (n=248) interacted with the feedback by clicking at least 1 link or modal. Descriptively, there was no difference between the feedback engagement across feedback arms (see the study by Kohlmann et al [12] for the results per study arm). At 1 month, 976 participants provided follow-up data of outcome measures (loss to follow-up: 17%), of whom 909 were included in the per protocol analysis. At 6 months, 965 participants provided follow-up data of outcome measures (loss to follow-up: 18%), of whom 902 were included in the per protocol analysis. The numbers per study arm and analysis time points are shown in the CONSORT flowchart (Figure 2).

Relevant demographic and clinical characteristics of the per protocol sample were balanced across the 3 study arms (Table 1). The mean participant age was 37.3 (SD 14.1) years, 72% (685/948) of the participants were women, and 52% (488/948) of the participants had a high education level. At baseline, the average PHQ-9 depression severity score was 14.8 (SD 4.0), the average score in emotional response to depressive symptoms was 7 (SD 1.9), 48% (455/948) of the participants reported to experience suicidal ideation at least several days within the last 2 weeks, and 87% (820/948) of the participants thought that they currently experienced or maybe experienced depression. Out of 820 participants who were reached for diagnostic telephone interviews, 63% (n=514) met the criteria for a major depressive disorder according to the *DSM-V*. Conversely, 37% (n=306) of the participants were classified as false-positive screens. Characteristics of the per protocol sample are comparable with those of the ITT sample (Multimedia Appendix 6).

**Table 1:** Baseline demographic and clinical characteristics of the per protocol sample.

			Total sample (n=948)		Nontailored feedback (n=314)		Tailored feedback (n=307)		No feedback (n=327)
Age (years), mean (SD)			37.3 (14)		37.8 (14)		36.8 (14.3)		37.2 (14)
**Sex, n (%)**									
	Female	685 (72)		223 (71)		219 (71)		243 (74)	
	Male	255 (27)		88 (28)		85 (28)		82 (25)	
	Divers	8 (0.8)		3 (1.0)		3 (1.0)		2 (0.6)	
German mother tongue, n (%)			902 (95)		295 (94)		296 (96)		311 (95)
Migration background, n (%)			103 (11)		30 (10)		32 (10)		41 (13)
Being in a relationship, n (%)			445 (47)		162 (52)		143 (47)		140 (43)
Living with others, n (%)			631 (67)		217 (69)		202 (66)		212 (65)
**Formal school education, n (%)**									
	Low (<10 years)	160 (17)		60 (19)		44 (15)		56 (17)	
	Middle (at least 10 years)	300 (32)		100 (32)		102 (33)		98 (30)	
	High (A-level or above)	488 (52)		154 (49)		161 (52)		173 (53)	
Working, n (%)			691 (73)		230 (73)		232 (76)		229 (70)
Depression severity (PHQ-9^a^), mean (SD)			14.8 (4)		14.9 (4.2)		14.6 (3.8)		14.8 (4)
Emotional response to depressive symptoms (composite scale), mean (SD)			7 (1.9)		7 (1.9)		6.9 (1.7)		6.9 (2)
Quality of life (EQ-5D-5L VAS^b^), mean (SD)			57.6 (21.6)		57.2 (21.6)		58.2 (21.3)		57.4 (21.9)
Anxiety severity (GAD-7^c^), mean (SD)			12.1 (4.3)		12.5 (4.2)		11.8 (4.3)		12 (4.3)
Somatic symptom severity (SSS-8^d^), mean (SD)			14.4 (5.2)		14.5 (5.1)		14.2 (5.2)		14.4 (5.2)
Depression risk factors^e^ (n), mean (SD)			6 (2.4)		6.1 (2.5)		5.9 (2.3)		6.1 (2.5)
**Frequency of suicidal ideation within last 2 weeks (PHQ-9 item 9), n (%)**									
	None	493 (52)		161 (51)		165 (54)		167 (51)	
	Several days	305 (32)		113 (36)		94 (31)		98 (30)	
	More than half the days	86 (9)		23 (7)		26 (9)		37 (11)	
	Nearly every day	64 (7)		17 (5)		22 (7)		25 (8)	
**Self-identifying as experiencing depression, n (%)**									
	No	128 (14)		32 (10)		51 (17)		45 (14)	
	Maybe	432 (46)		160(51)		141 (46)		131 (40)	
	Yes	388 (41)		122 (39)		115 (38)		151 (46)	
	Meeting criteria for major depressive disorder (SCID^f^)	514 (63)^g^		161 (61)^h^		172 (64)^i^		181 (62)^j^	

^a^PHQ-9: Patient Health Questionnaire-9 (0-27).

^b^VAS: visual analogue scale (0-100).

^c^GAD-7: Generalized Anxiety Disorder-7 (0-21).

^d^SSS-8: Somatic Symptom Scale (0-32).

^e^Risk factors included self-reported anxiety, addiction, traumatic life events, persistent physical symptoms, mood swings, chronic physical condition, lack of social support, mental comorbidity, mental comorbidity in family, history of suicide, current pregnancy, postnatal phase, menopause, and premenstrual syndrome.

^f^SCID: Structured Clinical Interview for *DSM-V* Disorders.

^g^A total of 128 cases with missing data.

^h^A total of 37 cases with missing data.

^i^A total of 51 cases with missing data.

^j^A total of 40 cases with missing data.

### Negative Effects Outcomes

**Figure 3:**
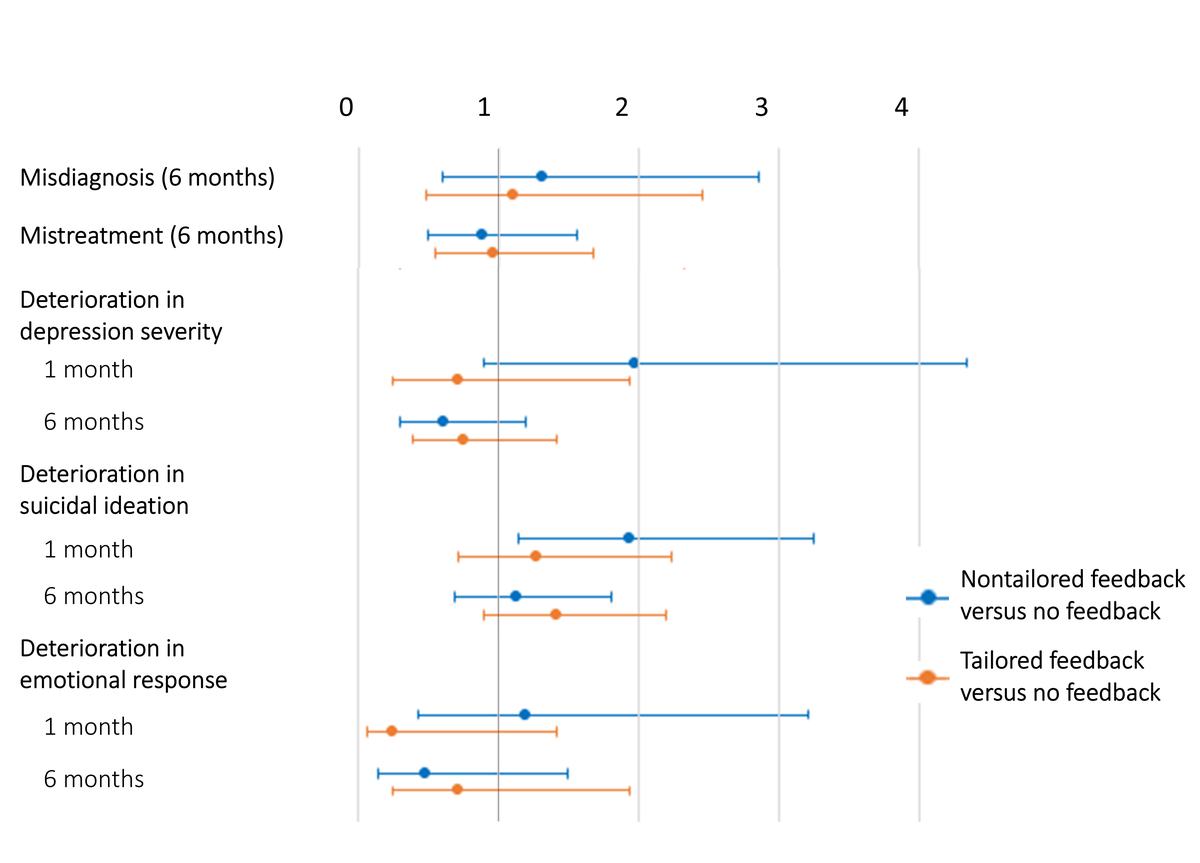
Relative risks (95% CIs) for all negative effects at all time points in the nontailored and tailored feedback arms as compared with no feedback.

Misdiagnosis rates 6 months after randomization were not higher after nontailored (RR 1.30, 95% CI 0.59-2.86; *P=*.51) or tailored feedback (RR 1.09, 95% CI 0.48-2.46; *P=*.84) as compared with no feedback, with rates of 4.9%, 4.1%, and 3.5% in the nontailored, the tailored, and the no feedback arm, respectively. Mistreatment rates 6 months after randomization were not higher after nontailored (RR 0.87, 95% CI 0.49-1.56; *P=*.65) or tailored feedback (RR 0.95, 95% CI 0.54-1.67; *P=*.86) either, with rates of 7.2%, 7.7%, and 8.3% in the nontailored, the tailored, and the no feedback arm. Rates of deterioration in depression severity were not higher after nontailored (1 month: RR 1.96, 95% CI 0.89-4.34; *P=*.10; 6 months: RR 0.60, 95% CI 0.3-1.19; *P=*.14) or tailored feedback (1 month: RR 0.70, 95% CI 0.25-1.94; *P=*.49; 6 months: RR 0.74, 95% CI 0.39-1.41; *P=*.37), with rates of 5.7%, 2.0%, and 2.9% at 1 month and 4.1%, 5.1%, and 6.8% at 6 months in the nontailored, tailored, and no feedback study arms. Rates of deterioration in emotional response to depressive symptoms were not higher after nontailored (1 month: RR 1.18, 95% CI 0.43-3.21; *P=*.75; 6 months: RR 0.46, 95% CI 0.14-1.49; *P=*.20) or tailored feedback (1 month: RR 0.23, 95% CI 0.06-1.42; *P=*.13; 6 months: RR 0.70, 95% CI 0.25-1.94; *P=*.49) either, with rates of 2.7%, 0.7%, and 2.3% at 1 month and 1.4%, 2.0%, and 2.9% at 6 months. Rates of deterioration in suicidal ideation were not higher after nontailored (RR 1.12, 95% CI 0.69-1.8; *P=*.66) or tailored feedback (RR 1.40, 95% CI 0.39-1.41; *P=*.15) at 6 months, with rates of 10.5%, 13.1%, and 9.4%. At 1 month, however, the rate of deterioration in suicidal ideation was almost 2-fold higher in the nontailored (RR 1.92, 95% CI 1.14-3.24; *P=*.01) but not in the tailored feedback arm (RR 1.26, 95% CI 0.25-1.94; *P=*.43), as compared with no feedback. Rates in the nontailored, the tailored, and the no feedback arms were 12.3%, 8.1%, and 6.4%. Absolute frequencies and rates for all negative effects per study arm and time point are shown in Table 2. Relative risks with corresponding 95% CIs are illustrated in Figure 3.

Results did not differ for the subgroup of false positives (*P*_interaction_ ranging between .29 and .80). Sensitivity analyses based on logistic regression models, as well as those in the ITT sample with the full analysis set and with missing data imputation based on the best-case scenario, showed comparable results. In the ITT analysis based on the worst case scenario, however, the RR for deterioration in suicidal ideation in the nontailored feedback arm at 1 month was not higher than that in the no feedback arm (RR 1.26, 95% CI 0.99-1.61; *P=*.07; Multimedia Appendix 7). Based on post hoc analyses, baseline demographic and clinical characteristics of all participants deteriorated in any outcome at any time point were comparable with the total sample (Multimedia Appendix 8).

**Table 2:** Absolute frequencies and rates of negative effects per study arm and time point.

			Participants		Nontailored feedback		Participants		Tailored feedback		Participants		No feedback
Misdiagnosis (6 months)^a^, n (%)			263		13 (4.9)		267		11 (4.1.)		290		11 (3.5)
**Mistreatment (6 months)^a^, n (%)**			263		19 (7.2)		267		21 (7.7)		290		24 (8.3)
	Psychotherapy^a^	263		13 (4.9)		267		17 (6.4)		290		18 (6.2)	
	Medication^a^	263		9 (3.4)		267		6 (2.2)		290		8 (2.8)	
**Deterioration in depression severity, n (%)**													
	1 month	300		17 (5.7)		297		6 (2.0)		312		9 (2.9)	
	6 months	296		12 (4.1)		297		15 (5.1)		309		21 (6.8)	
**Deterioration in suicidal ideation, n (%)**													
	1 month	300		37 (12.3)^b^		297		24 (8.1)		312		20 (6.4)	
	6 months	296		31 (10.5)		297		39 (13.1)		309		29 (9.4)	
**Deterioration in emotional response, n (%)**													
	1 month	299		8 (2.7)		296		2 (0.7)		308		7 (2.3)	
	6 months	294		4 (1.4)		299		6 (2)		307		9 (2.9)	

^a^Participants who completed both the follow-up assessment and the Structured Clinical Interview for *DSM-V* Disorders depression module at baseline.

^b^Significantly increased relative risk as compared with no feedback, with *P*<.05.

## Discussion

To the best of our knowledge, this secondary analysis is the first study to systematically examine potential negative effects of feedback after web-based depression screening in a large sample of currently undiagnosed and untreated individuals with at least moderate depression severity.

### Principal Findings

The results indicate that feedback, both nontailored and tailored, was not associated with increased rates of misdiagnosis, mistreatment, deterioration in depression severity, or deterioration in emotional response to symptoms as compared with no feedback. Deterioration of suicidal ideation, however, appeared to be more likely 1 month after receiving nontailored feedback, as compared with no feedback. Although almost 40% of the sample turned out to be screened false positive, irrespective of the study arm, rates of subsequent misdiagnosis and mistreatment were lower than 5% and 9%, respectively, with rates of pharmacotherapy ranging even lower than 4%. Across study arms, deterioration in emotional response to depressive symptoms was reported by at most 3% of participants, deterioration in depression severity by at most 7% of participants, and deterioration of suicidal ideation by at most 13% of participants.

### Comparison With Prior Work

There are 3 main findings that contribute to the scientific debate on negative effects of web-based depression screening outlined in the Introduction section. First, the results regarding mistreatment and misdiagnosis emphasize that feedback after web-based depression screening is not associated with inadequate management and care for individuals who receive false-positive feedback—even when the rate of false positives is relatively high and when the feedback refers to a health system that covers depression care as it is true for Germany. These results extend on prior findings that feedback after web-based depression screening does not affect service uptake [12,43]. Furthermore, they refute the most prominent but opinion-based criticism against web-based depression screening [4,14].

Second, there is also no indication that feedback after web-based depression screening induces negative psychological effects such as deterioration in depression severity and emotional response to symptoms. Notably, the rates for deterioration in depression severity of at most 7% found in this study are comparable with those reported in care-as-usual conditions in psychotherapy trials [45]. The null findings regarding deterioration in emotional response to symptoms, however, appear to conflict with prior qualitative evidence suggesting that web-based depression screening does induce negative emotions and distress in some individuals [8,18]. An explanation for this discrepancy might be that negative emotional effects might be induced not only by the feedback but also by the screening questions alone, which has been reported in a qualitative follow-up study of the DISCOVER trial [18]. Furthermore, it might be that the construct emotional response, defined by items assessing concern and emotional affectedness about the symptoms, relates more to a cognitive evaluation of symptoms rather than capturing an actual emotional state. Therefore, assessing outcomes such as distress or negative affectivity shortly after providing screening and comparing these between a screening only and a feedback condition appear worthwhile to further address these issues (see the studies by Gould et al [46] and Robinson et al [47] for exemplary study designs in suicide screening).

Third, the current results indicate that nontailored feedback, in contrast to tailored feedback, might lead to increased suicidal ideation after 1 month. This finding is contradictory to results from a randomized clinical trial on screening and feedback in the primary care setting [48] but in line with prior observational evidence regarding web-based screening [7]. Explanations for such an effect might be that receiving a diagnosis on the web might induce hopelessness, a known risk factor for suicidal ideation [49], or that the referral initiation process may be overwhelming, thereby triggering decompensation [7]. However, it remains an open question why nontailored but not tailored feedback should increase suicidal ideation: against our hypothesis, neither the usage of the feedback nor any other outcome differed between the 2 feedback arms [12].

Notably, when qualitatively asked in follow-up telephone interviews 6 months after screening, only 1% (9/909) of the participants retrospectively reported negative effects attributed to trial participation (see the study by Kohlmann et al [12]). Explanations for the discrepancy between this low rate and present negative effects rates of up to 13% might be that in qualitative interviews, negative effects might have been stigmatizing to report (eg, in the case of suicidal ideation), might not be remembered retrospectively, or might subjectively not be classified as an adverse event by participants.

### Limitations

The interpretation of the present results should be considered in the context of the study’s limitations. First, this secondary analysis of the DISCOVER trial was planned post hoc and therefore not powered to detect differences between study arms regarding the selected outcomes. Indeed, rates of negative effects turned out to be low (with a maximum of 39 participants per study arm), wherefore analyses may be underpowered to detect significant effects. On the other hand, multiple testing might have led to overestimation of significance in the case of deterioration in suicidal ideation. To robustly examine negative effects in the future, trials with higher sample sizes that prospectively address negative effects are needed.

Second, the underlying DISCOVER trial did not explicitly call for individuals seeking depression screening. As these may be more eager to follow the advice of the feedback, in the current sample, misdiagnosis and mistreatment might be underestimated as compared with individuals using public depression screening tools. To increase the generalizability of results, future studies should target the recruitment of participants who actively seek web-based depression screening.

Third, as this is a secondary analysis, outcome selection was limited. Although the existing data allowed for assessing a range of relevant negative effects, future research should consider including further outcomes such as distress, suicidal behavior, stigma, treatment side effects, or overdiagnosis (ie, the diagnosis of correctly diagnosed but mild cases that would not benefit from treatment [16]). Furthermore, the present outcomes are based on self-reports and would benefit from more objective data (eg, for misdiagnosis) from health care providers. Regarding misdiagnosis and mistreatment, it cannot be ruled out that participants (correctly) received a burnout diagnosis or antidepressant medication or psychotherapy for conditions other than depressive disorders, wherefore rates may be overestimated. Furthermore, the operationalizations of suicidal ideation and emotional response to depressive symptoms are based on a single item and a composite score, respectively, that are not well validated for this purpose (see the studies by Na et al [50] and Rossom et al [51] for research on the validity of the PHQ-9 suicide item). Future research on negative effects should use valid and reliable measures to assess these outcomes (see the study by Erford et al [52] for a review on suicide ideation assessment instruments).

Notably, the findings refer to the German health care system, where psychotherapy is available and covered by the social health insurance. Particularly, rates for misdiagnosis and mistreatment might differ in other countries with differing health policies.

### Future Directions

Given that the current results should be interpreted with caution due to the study’s limitations, more robust research is needed to further address negative effects in web-based depression screening. Particularly, prospective and well-powered trials that validly assess suicidal ideation, preferably directly after the provision of screening and feedback, are needed (see the studies by Gould et al [46] and Robinson et al [47] for exemplary study designs in suicide screening). If future studies corroborate an association of web-based screening and feedback with suicidal ideation, this finding needs to inform regulations of currently unmonitored web-based depression tests. Furthermore, the findings should also inform research regarding comparable depression screening in medical and primary care settings, which is currently recommended in many countries despite very uncertain evidence regarding potential harms [53].

### Conclusions

The results of this secondary analysis indicate that feedback after web-based depression screening is neither associated with health care–related negative effects such as misdiagnosis and mistreatment nor with psychological negative effects such as deterioration in depression severity or emotional response to symptoms. However, it cannot be ruled out that nontailored feedback may be associated with increased suicidal ideation. Against the background of the study’s secondary design, robust prospective research on negative effects and particularly suicidal ideation in web-based depression screening is needed to inform current practice of public web-based depression screening as well as research in the field of depression screening in general.

## Data Availability

Individual participant data that underlie the results reported in this paper, after deidentification (text, tables, figures, and appendices), will be shared by the corresponding author upon reasonable request for academic and research purposes and subject to data-sharing agreements.

## Appendix

Multimedia Appendix 1 Illustration of the digitized 9-item Patient Health Questionnaire as displayed to study participants.

Multimedia Appendix 2 Illustrations of complete nontailored feedback.

Multimedia Appendix 3 Illustrations of complete tailored feedback.

Multimedia Appendix 4 Illustration of suicidal ideation feedback.

Multimedia Appendix 5 CONSORT-eHEALTH checklist (V 1.6.2).

Multimedia Appendix 6 Characteristics of intention-to-treat sample.

Multimedia Appendix 7 Sensitivity analyses.

Multimedia Appendix 8 Post hoc analyses.

## Abbreviations

CONSORT: Consolidated Standards of Reporting Trials
DSM-V: Diagnostic and Statistical Manual of Mental Disorders (Fifth Edition)
IPQ: Illness Perception Questionnaire
ITT: intention-to-treat
PHQ-9: 9-item Patient Health Questionnaire
RCI: reliable change index
RR: relative risk
SCID: Structured Clinical Interview for DSM-V Disorders
