# Supplementary material for: Potential Harms of Feedback After Web-Based Depression Screening: Secondary Analysis of Negative Effects in the Randomized Controlled DISCOVER Trial
Source: J Med Internet Res. 2025 Apr 30;27:e59476. doi: 10.2196/59476 (PMC12079080; doi:10.2196/59476)
Supplement: Multimedia Appendix 1 [file jmir_v27i1e59476_app1.pptx]

## Slide 1
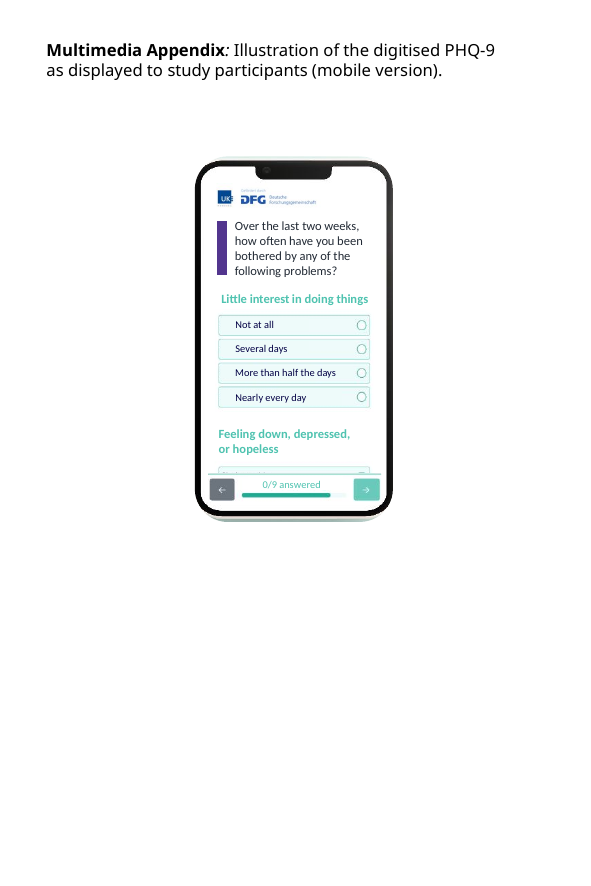

Multimedia Appendix: Illustration of the digitised PHQ-9 as displayed to study participants (mobile version).
Over the last two weeks, how often have you been bothered by any of the following problems?
Little interest in doing things
Not at all
Several days
More than half the days
Nearly every day
Feeling down, depressed, or hopeless
0/9 answered
