# Supplementary material for: Potential Harms of Feedback After Web-Based Depression Screening: Secondary Analysis of Negative Effects in the Randomized Controlled DISCOVER Trial
Source: J Med Internet Res. 2025 Apr 30;27:e59476. doi: 10.2196/59476 (PMC12079080; doi:10.2196/59476)
Supplement: Multimedia Appendix 2 [file jmir_v27i1e59476_app2.pptx]

## Slide 1
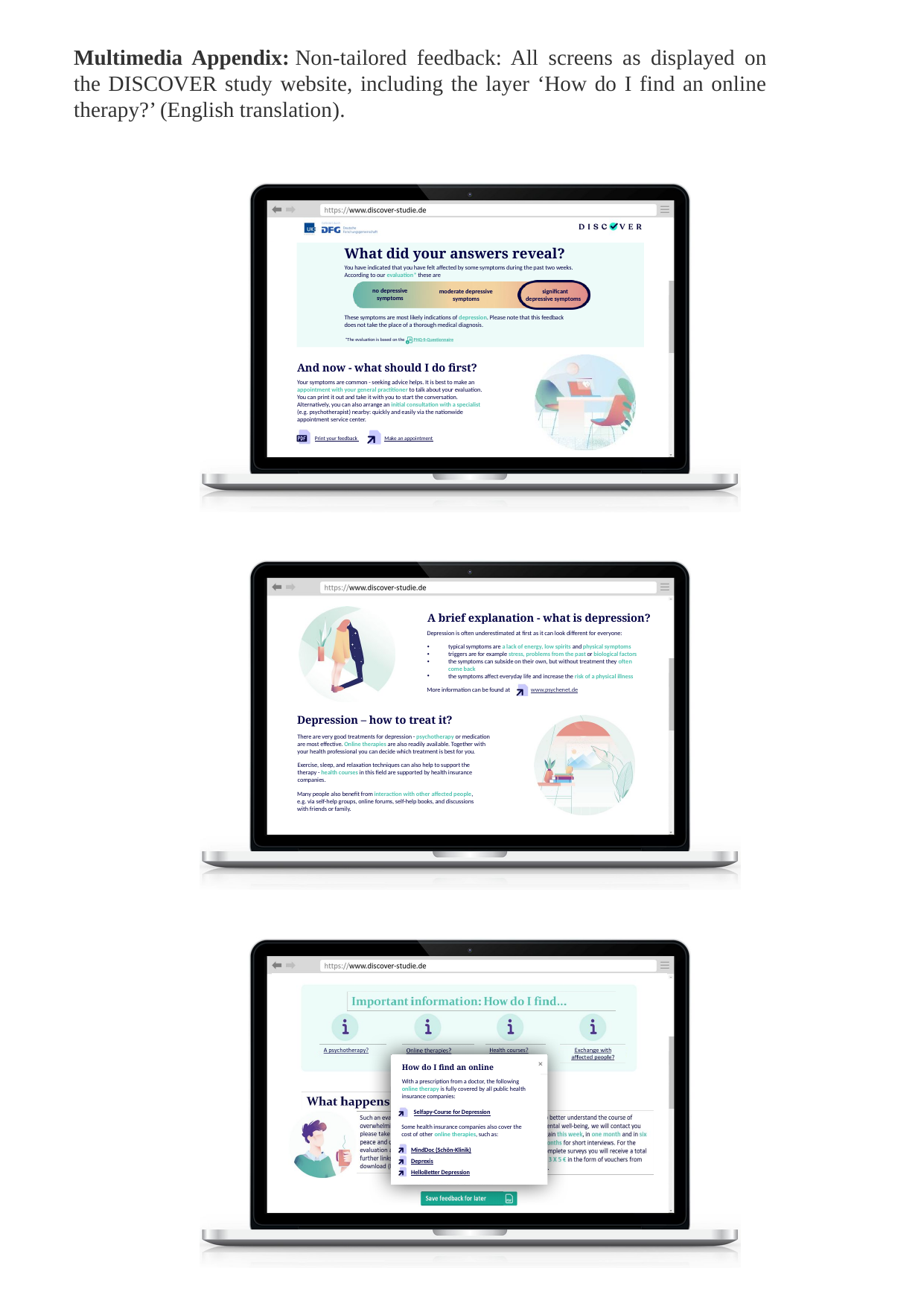

Multimedia Appendix: Non-tailored feedback: All screens as displayed on the DISCOVER study website, including the layer ‘How do I find an online therapy?’ (English translation).
You have indicated that you have felt affected by some symptoms during the past two weeks.
According to our evaluation* these are
What did your answers reveal?
no depressive symptoms
moderate depressive symptoms
 significant depressive symptoms
These symptoms are most likely indications of depression. Please note that this feedback does not take the place of a thorough medical diagnosis.
*The evaluation is based on the
And now - what should I do first?
Your symptoms are common - seeking advice helps. It is best to make an appointment with your general practitioner to talk about your evaluation. You can print it out and take it with you to start the conversation. Alternatively, you can also arrange an initial consultation with a specialist (e.g. psychotherapist) nearby: quickly and easily via the nationwide appointment service center.
Print your feedback
Make an appointment
A brief explanation - what is depression?
Depression is often underestimated at first as it can look different for everyone:
typical symptoms are a lack of energy, low spirits and physical symptoms
triggers are for example stress, problems from the past or biological factors
the symptoms can subside on their own, but without treatment they often come back
the symptoms affect everyday life and increase the risk of a physical illness
www.psychenet.de
More information can be found at
Depression – how to treat it?
There are very good treatments for depression - psychotherapy or medication are most effective. Online therapies are also readily available. Together with your health professional you can decide which treatment is best for you.
Exercise, sleep, and relaxation techniques can also help to support the therapy - health courses in this field are supported by health insurance companies.
Many people also benefit from interaction with other affected people, e.g. via self-help groups, online forums, self-help books, and discussions with friends or family.
How do I find an online therapy?
With a prescription from a doctor, the following online therapy is fully covered by all public health insurance companies:
Selfapy-Course for Depression
Some health insurance companies also cover the cost of other online therapies, such as:
MindDoc (Schön-Klinik)
Deprexis
HelloBetter Depression
