# Supplementary material for: Potential Harms of Feedback After Web-Based Depression Screening: Secondary Analysis of Negative Effects in the Randomized Controlled DISCOVER Trial
Source: J Med Internet Res. 2025 Apr 30;27:e59476. doi: 10.2196/59476 (PMC12079080; doi:10.2196/59476)
Supplement: Multimedia Appendix 3 [file jmir_v27i1e59476_app3.pptx]

## Slide 1
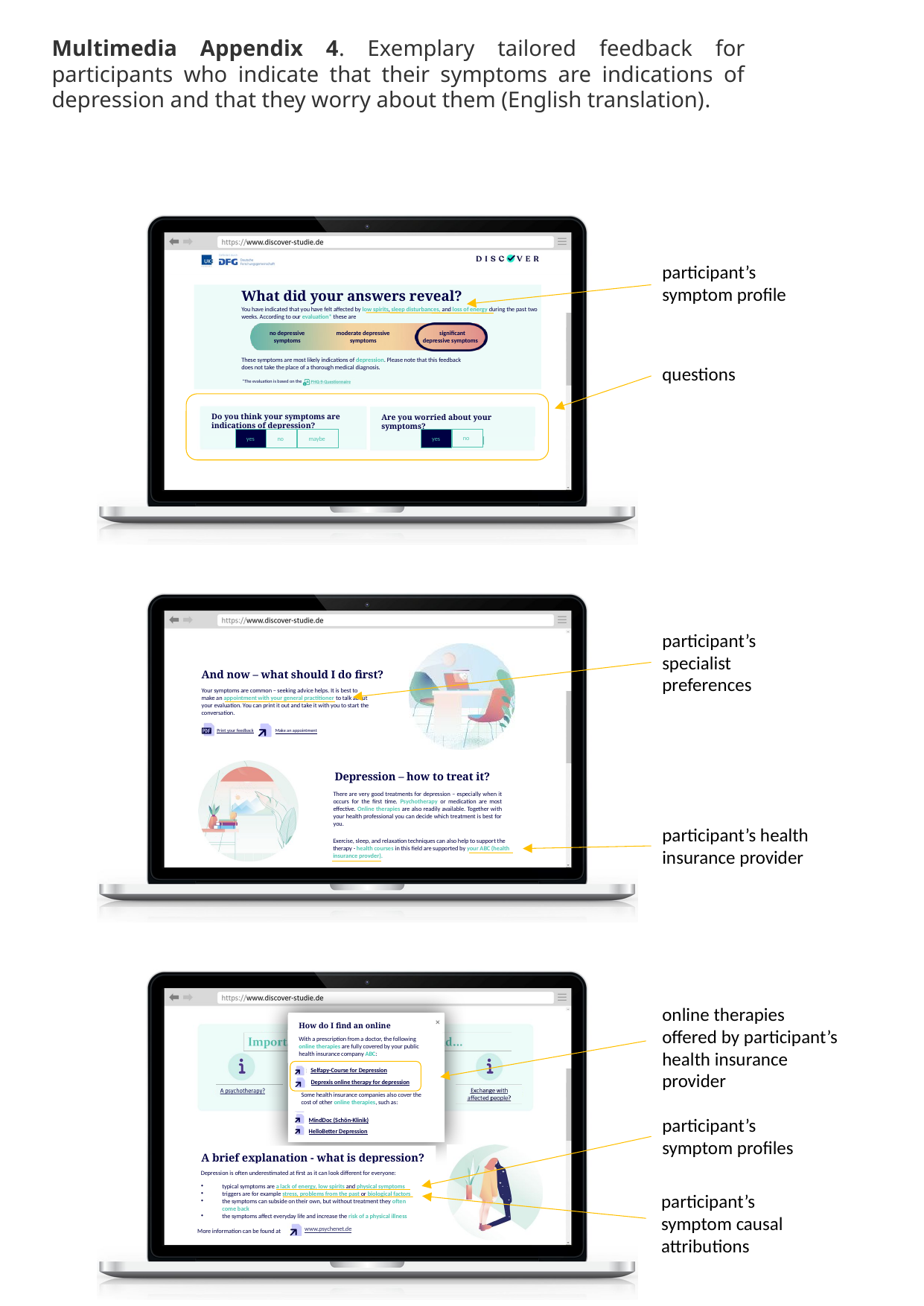

Multimedia Appendix 4. Exemplary tailored feedback for participants who indicate that their symptoms are indications of depression and that they worry about them (English translation).
participant’s symptom profile
You have indicated that you have felt affected by low spirits, sleep disturbances, and loss of energy during the past two weeks. According to our evaluation* these are
What did your answers reveal?
no depressive symptoms
moderate depressive symptoms
 significant depressive symptoms
questions
These symptoms are most likely indications of depression. Please note that this feedback does not take the place of a thorough medical diagnosis.
*The evaluation is based on the
Do you think your symptoms are indications of depression?
Are you worried about your symptoms?
no
yes
maybe
yes
no
participant’s specialist preferences
And now – what should I do first?
Your symptoms are common – seeking advice helps. It is best to make an appointment with your general practitioner to talk about your evaluation. You can print it out and take it with you to start the conversation.
Print your feedback
Make an appointment
 Depression – how to treat it?
There are very good treatments for depression – especially when it occurs for the first time. Psychotherapy or medication are most effective. Online therapies are also readily available. Together with your health professional you can decide which treatment is best for you.
participant’s health insurance provider
Exercise, sleep, and relaxation techniques can also help to support the therapy - health courses in this field are supported by your ABC (health insurance provder).
online therapies offered by participant’s health insurance provider
How do I find an online therapy?
With a prescription from a doctor, the following online therapies are fully covered by your public health insurance company ABC:
Selfapy-Course for Depression
Deprexis online therapy for depression
Some health insurance companies also cover the cost of other online therapies, such as:
MindDoc (Schön-Klinik)
HelloBetter Depression
participant’s symptom profiles
A brief explanation - what is depression?
Depression is often underestimated at first as it can look different for everyone:
typical symptoms are a lack of energy, low spirits and physical symptoms
triggers are for example stress, problems from the past or biological factors
the symptoms can subside on their own, but without treatment they often come back
the symptoms affect everyday life and increase the risk of a physical illness
participant’s symptom causal attributions
www.psychenet.de
More information can be found at

## Slide 2
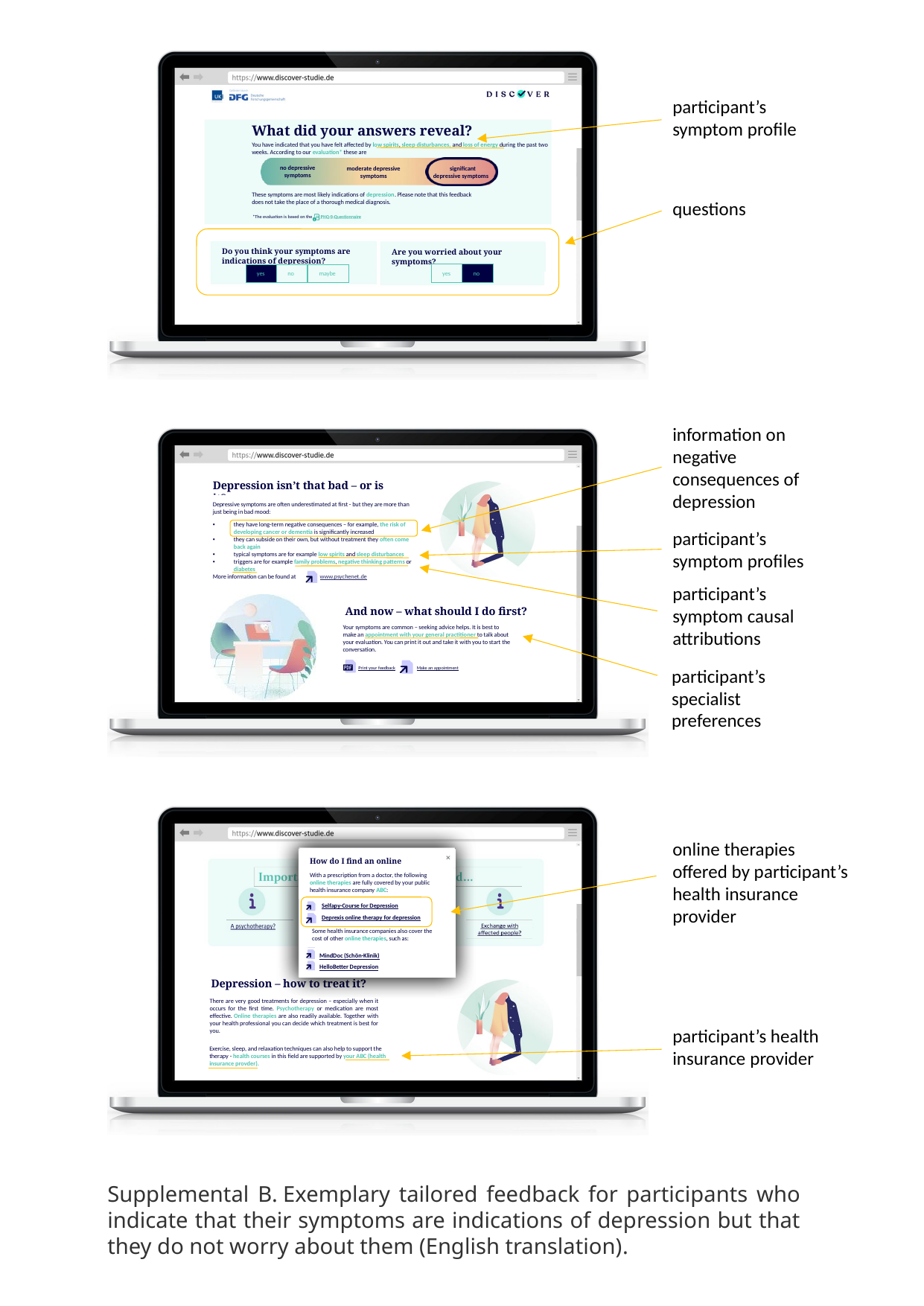

participant’s symptom profile
You have indicated that you have felt affected by low spirits, sleep disturbances, and loss of energy during the past two weeks. According to our evaluation* these are
What did your answers reveal?
no depressive symptoms
moderate depressive symptoms
 significant depressive symptoms
questions
These symptoms are most likely indications of depression. Please note that this feedback does not take the place of a thorough medical diagnosis.
*The evaluation is based on the
Do you think your symptoms are indications of depression?
Are you worried about your symptoms?
no
yes
maybe
yes
no
information on negative consequences of depression
Depression isn’t that bad – or is it?
Depressive symptoms are often underestimated at first - but they are more than just being in bad mood:
they have long-term negative consequences – for example, the risk of developing cancer or dementia is significantly increased
they can subside on their own, but without treatment they often come back again
typical symptoms are for example low spirits and sleep disturbances
triggers are for example family problems, negative thinking patterns or diabetes
More information can be found at
participant’s symptom profiles
www.psychenet.de
participant’s symptom causal attributions
 And now – what should I do first?
Your symptoms are common – seeking advice helps. It is best to make an appointment with your general practitioner to talk about your evaluation. You can print it out and take it with you to start the conversation.
participant’s specialist preferences
Print your feedback
Make an appointment
online therapies offered by participant’s health insurance provider
How do I find an online therapy?
How do I find an online therapy?
With a prescription from a doctor, the following online therapies are fully covered by your public health insurance company ABC:
With a prescription from a doctor, the following online therapies are fully covered by your public health insurance company ABC:
Selfapy-Course for Depression
Selfapy-Course for Depression
Deprexis online therapy for depression
Deprexis online therapy for depression
Some health insurance companies also cover the cost of other online therapies, such as:
Some health insurance companies also cover the cost of other online therapies, such as:
MindDoc (Schön-Klinik)
HelloBetter Depression
MindDoc (Schön-Klinik)
HelloBetter Depression
 Depression – how to treat it?
There are very good treatments for depression – especially when it occurs for the first time. Psychotherapy or medication are most effective. Online therapies are also readily available. Together with your health professional you can decide which treatment is best for you.
participant’s health insurance provider
Exercise, sleep, and relaxation techniques can also help to support the therapy - health courses in this field are supported by your ABC (health insurance provder).
Supplemental B. Exemplary tailored feedback for participants who indicate that their symptoms are indications of depression but that they do not worry about them (English translation).

## Slide 3
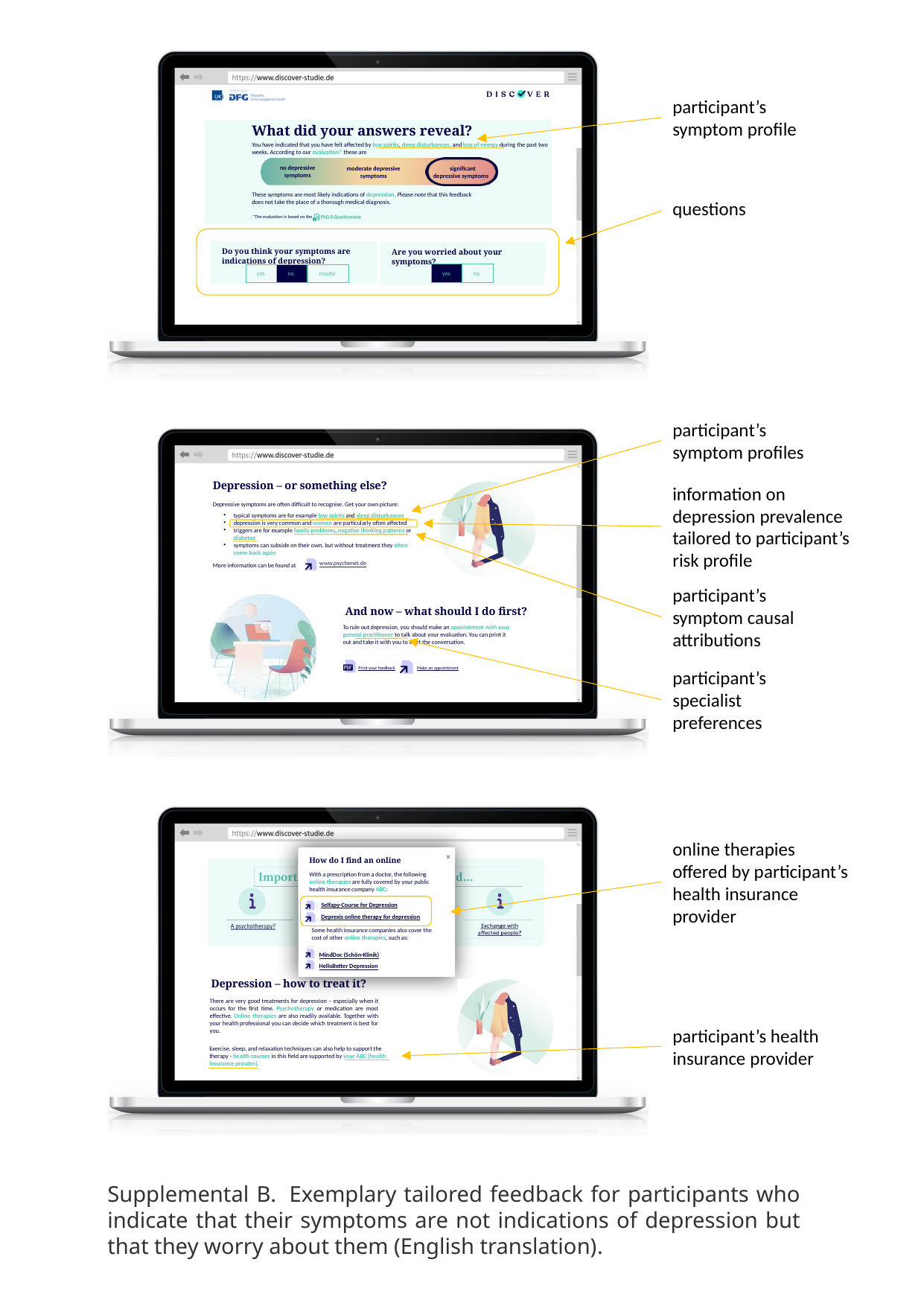

participant’s symptom profile
You have indicated that you have felt affected by low spirits, sleep disturbances, and loss of energy during the past two weeks. According to our evaluation* these are
What did your answers reveal?
no depressive symptoms
moderate depressive symptoms
 significant depressive symptoms
questions
These symptoms are most likely indications of depression. Please note that this feedback does not take the place of a thorough medical diagnosis.
*The evaluation is based on the
Do you think your symptoms are indications of depression?
Are you worried about your symptoms?
no
yes
maybe
yes
no
participant’s symptom profiles
Depression – or something else?
information on depression prevalence tailored to participant’s risk profile
Depressive symptoms are often difficult to recognise. Get your own picture:
typical symptoms are for example low spirits and sleep disturbances
depression is very common and women are particularly often affected
triggers are for example family problems, negative thinking patterns or diabetes
symptoms can subside on their own, but without treatment they often come back again
More information can be found at
www.psychenet.de
participant’s symptom causal attributions
 And now – what should I do first?
To rule out depression, you should make an appointment with your general practitioner to talk about your evaluation. You can print it out and take it with you to start the conversation.
Print your feedback
Make an appointment
participant’s specialist preferences
online therapies offered by participant’s health insurance provider
How do I find an online therapy?
With a prescription from a doctor, the following online therapies are fully covered by your public health insurance company ABC:
Selfapy-Course for Depression
Deprexis online therapy for depression
Some health insurance companies also cover the cost of other online therapies, such as:
MindDoc (Schön-Klinik)
HelloBetter Depression
 Depression – how to treat it?
There are very good treatments for depression – especially when it occurs for the first time. Psychotherapy or medication are most effective. Online therapies are also readily available. Together with your health professional you can decide which treatment is best for you.
participant’s health insurance provider
Exercise, sleep, and relaxation techniques can also help to support the therapy - health courses in this field are supported by your ABC (health insurance provder).
Supplemental B.  Exemplary tailored feedback for participants who indicate that their symptoms are not indications of depression but that they worry about them (English translation).

## Slide 4
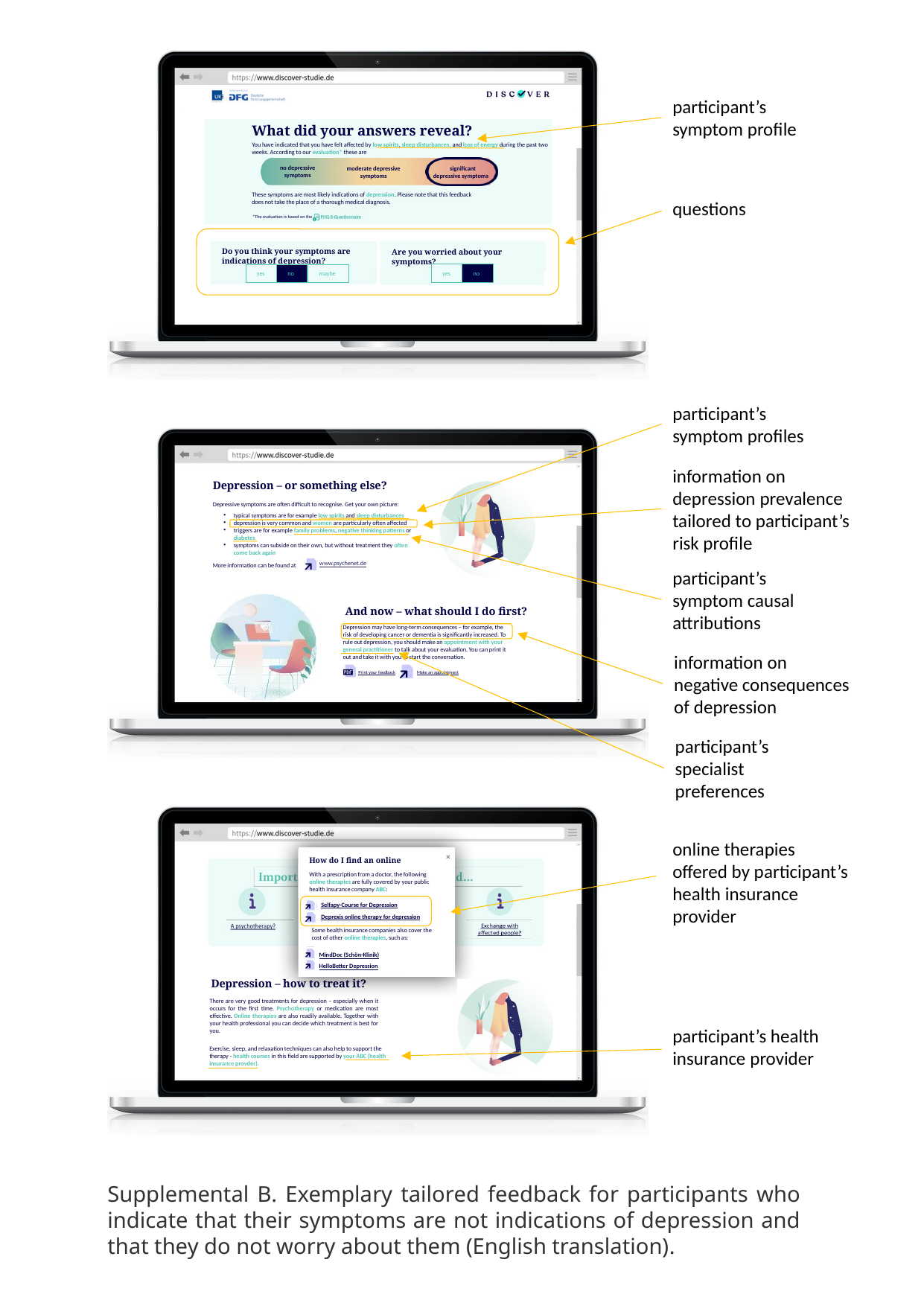

participant’s symptom profile
You have indicated that you have felt affected by low spirits, sleep disturbances, and loss of energy during the past two weeks. According to our evaluation* these are
What did your answers reveal?
no depressive symptoms
moderate depressive symptoms
 significant depressive symptoms
questions
These symptoms are most likely indications of depression. Please note that this feedback does not take the place of a thorough medical diagnosis.
*The evaluation is based on the
Do you think your symptoms are indications of depression?
Are you worried about your symptoms?
no
yes
maybe
yes
no
participant’s symptom profiles
information on depression prevalence tailored to participant’s risk profile
Depression – or something else?
Depressive symptoms are often difficult to recognise. Get your own picture:
typical symptoms are for example low spirits and sleep disturbances
depression is very common and women are particularly often affected
triggers are for example family problems, negative thinking patterns or diabetes
symptoms can subside on their own, but without treatment they often come back again
More information can be found at
www.psychenet.de
participant’s symptom causal attributions
 And now – what should I do first?
Depression may have long-term consequences – for example, the risk of developing cancer or dementia is significantly increased. To rule out depression, you should make an appointment with your general practitioner to talk about your evaluation. You can print it out and take it with you to start the conversation.
information on negative consequences of depression
Print your feedback
Make an appointment
participant’s specialist preferences
online therapies offered by participant’s health insurance provider
How do I find an online therapy?
With a prescription from a doctor, the following online therapies are fully covered by your public health insurance company ABC:
Selfapy-Course for Depression
Deprexis online therapy for depression
Some health insurance companies also cover the cost of other online therapies, such as:
MindDoc (Schön-Klinik)
HelloBetter Depression
 Depression – how to treat it?
There are very good treatments for depression – especially when it occurs for the first time. Psychotherapy or medication are most effective. Online therapies are also readily available. Together with your health professional you can decide which treatment is best for you.
participant’s health insurance provider
Exercise, sleep, and relaxation techniques can also help to support the therapy - health courses in this field are supported by your ABC (health insurance provder).
Supplemental B. Exemplary tailored feedback for participants who indicate that their symptoms are not indications of depression and that they do not worry about them (English translation).
