# Supplementary material for: Potential Harms of Feedback After Web-Based Depression Screening: Secondary Analysis of Negative Effects in the Randomized Controlled DISCOVER Trial
Source: J Med Internet Res. 2025 Apr 30;27:e59476. doi: 10.2196/59476 (PMC12079080; doi:10.2196/59476)
Supplement: Multimedia Appendix 4 [file jmir_v27i1e59476_app4.pptx]

## Slide 1
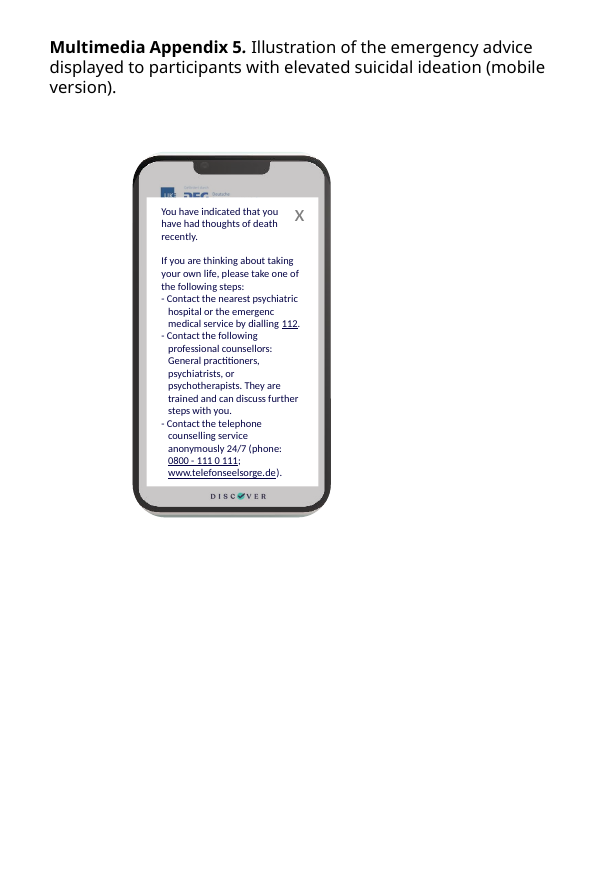

Multimedia Appendix 5. Illustration of the emergency advice displayed to participants with elevated suicidal ideation (mobile version).
Thank you for participating!
By taking part in D I S C O V E R you have made an important contribution to mental wellbeing research!
x
You have indicated that you
have had thoughts of death recently.
If you are thinking about taking your own life, please take one of the following steps:
- Contact the nearest psychiatric
 hospital or the emergenc
 medical service by dialling 112.
- Contact the following
 professional counsellors:
 General practitioners,
 psychiatrists, or
 psychotherapists. They are
 trained and can discuss further
 steps with you.
- Contact the telephone
 counselling service
 anonymously 24/7 (phone:
 0800 - 111 0 111;
 www.telefonseelsorge.de).
