# Supplementary material for: Potential Harms of Feedback After Web-Based Depression Screening: Secondary Analysis of Negative Effects in the Randomized Controlled DISCOVER Trial
Source: J Med Internet Res. 2025 Apr 30;27:e59476. doi: 10.2196/59476 (PMC12079080; doi:10.2196/59476)
Supplement: Multimedia Appendix 5 [file jmir_v27i1e59476_app5.pdf]

Multimedia Appendix: CONSORT-EHEALTH Checklist V1.6.2 Report

(based on CONSORT-EHEALTH V1.6.1 from: [CONSORT-EHEALTH (V 1.6.1) - Submission/Publication Form (google.com)](); Eysenbach G, CONSORT-EHEALTH GroupCONSORT-EHEALTH: Improving and Standardizing Evaluation Reports of Web-based and Mobile Health InterventionsJ Med Internet Res 2011;13(4):e126

Date completed // updated

17/2/2024 // 12/04/2024

by

Franziska Sikorski

Does feedback after internet-based depression screening cause harm? A secondary analysis of negative effects in the randomised controlled DISCOVER trial

Language

German

Accessibility; URL of your Intervention Website or App

exemplary access is possible (feedback without screening); https://www.discover-studie.de/rueckmeldung

Primary Medical Indication/Disease/Condition

undiagnosed depressive disorders

Primary Medical Indication/Disease/Condition

mistreatment, misdiagnosis, deterioration in depression severity, deterioration in emotional response to symptoms, deterioration in suicidal ideation

Overall, was the intervention effective?

potentially harmful: increased deterioration in suicidal ideation in tailored feedback arm

TITLE

1a) Does your paper address CONSORT item 1a (identification as a randomized trial in the title)?

yes

1a-i) Identify the mode of delivery in the title

"Does feedback after web-based depression screening cause harm? A secondary analysis of negative effects in the randomised controlled DISCOVER trial"

1a-ii) Non-web-based components or important co-interventions in title

This is not relevant to this manuscript.

1a-iii) Primary condition or target group in the title

The intervention is not addressing a specific condition (general population; with undiagnosed depressive symptoms).

ABSTRACT

1b-i) Key features/functionalities/components of the intervention and comparator in the METHODS section of the ABSTRACT

"Undiagnosed but affected individuals with a positive depression screening score (Patient Health Questionnaire-9, PHQ-9 ≥ 10 points) were randomised to receive no feedback, nontailored feedback, or tailored feedback on their screening result together with recommendations to seek diagnostic advice."

1b-ii) Level of human involvement in the METHODS section of the ABSTRACT

"We aimed to examine whether automated feedback after internet-based depression screening…"

1b-iii) Open vs. closed, web-based (self-assessment) vs. face-to-face assessments in the METHODS section of the ABSTRACT

"Participants were followed-up at one and six months via online questionnaires. Misdiagnosis and mistreatment were operationalised as having received a depression diagnosis by a health professional and as having started psychotherapy or antidepressant medication since screening while not meeting the DSM-5 criteria of a major depression (diagnostic telephone interviews). Deterioration in depressive symptoms was defined as a pre-post change of ≥ 4.4 points in the PHQ-9, deterioration in emotional response to symptoms as a pre-post change of ≥ 3.1 points in a composite scale based on the Brief Illness Perception Questionnaire, and deterioration in suicidal ideation as a pre-post change of ≥ 1 point in the PHQ-9 suicide item."

1b-iv) RESULTS section in abstract must contain use data

"In the per protocol sample of 948 participants who opened the feedback…"

1b-v) CONCLUSIONS/DISCUSSION in abstract for negative trials

Not applicable, as all participants used the intervention.

INTRODUCTION

2a-i) Problem and the type of system/solution

"Depressive disorders, although being among the most disabling and most prevalent disorders worldwide [1], often remain undetected and therefore untreated [2]. In the last decades, depression screening has been increasingly discussed as promising to reach those affected but undetected at an early stage: In addition to population level screening in routine clinical care, as for example recommended in the United States [3], advocates also speak out in favour of screening for depression online [4]. For many affected individuals, the internet is already the favoured source for information on mental health [5, 6]. Further, so called internet-based depression tests are widely promoted by mental health-related institutions and frequently used by those seeking diagnostic advice [7]. The rational of internet-based depression tests typically involves administering symptom-based screening questionnaires and providing individuals with direct feedback on screening results, sometimes supplemented by links or referrals to services. The feedback is thought to empower affected individuals to better act on their symptoms [8] and to seek diagnostic consultation and, if necessary, appropriate care. As such, it might improve early detection and management of depression."

2a-ii) Scientific background, rationale: What is known about the (type of) system

“Negative effects, if present, would therefore likely be genereated without creating substantial health benefits. Evidence in this regard is, however, scarce, with the current scientific debate being mainly reflected by opinion papers: The first area of negative effects of depression screening, discussed in both medical and internet-based contexts, relates to inadequate management and care for individuals who receive false positive feedback. Critics particularly point to the risk of increased rates of misdiagnosis and mistreatment following screening. This, again, is assumed to lead to unnecessary iatrogenic effects such as adverse medication and psychotherapy side effects in healthy individuals, societal costs, and waste of limited health care resources resulting in potential undertreatment of more severe cases [4, 12, 13]. A second area of concern relates to negative psychological effects to the feedback of screening results. It is assumed that the associated labeling, resembling a clinical diagnosis, might induce anxiety, distress, stigma, or nocebo effects such as for example deterioration of symptoms [4, 13, 14]. These effects could be amplified by the fact that, in contrast to medical settings, in internet-based depression screening the ‘diagnosis’ would be delivered without a health professional who could provide emotional support or advice on further steps [15]. Indeed, in qualitative studies on internet-based mental health screening some participants describe having been discouraged, shocked or concerned by the feedback they received [8, 16]. Further, one observational study found that screening procedures including

referrals to in-person care had a higher likelihood of subsequent online searches for suicidal intent, potentially suggesting a deterioration of suicidal ideation [17]."

Does your paper address CONSORT subitem 2b?

"In the present study, we addressed this lack of evidence by analysing data from our recently conducted randomised controlled trial on the efficacy of feedback after internet-based depression screening (cite paper, when published). Based on the potential negative effects discussed in the literature and on outcomes assessed in that trial, we aimed to examine whether feedback after internet-based depression screening is associated with increased misdiagnosis, mistreatment, deterioration in depressive symptoms, deterioration in emotional response to symptoms, and deterioration in suicidal ideation one and six months after the screening."

METHODS

3a) CONSORT: Description of trial design (such as parallel, factorial) including allocation ratio

"DISCOVER was an investigator-initiated, observer-blinded, three-armed, randomised controlled trial that compared automated feedback with no feedback after internet-based depression screening. After being screened for depression with the digitised Patient Health Questionnaire-9 (PHQ-9 [25]), eligible participants were randomised to receive either no feedback, nontailored feedback or tailored feedback on their screening result (1:1:1 allocation ratio)."

3b) CONSORT: Important changes to methods after trial commencement (such as eligibility criteria), with reasons

"We conducted small deviations from the preregistration: we added the outcomes misdiagnosis and emotional response to symptoms, as we deemed this of clinical interest. Further, we added sensitivity analyses based on logistic regression models."

3b-i) Bug fixes, Downtimes, Content Changes

We did not have major bugs or down time for this trial.

4a) CONSORT: Eligibility criteria for participants

"Participants were individuals aged 18 years or above with at least moderate depression severity (PHQ-9 $\geq$ 10) but not diagnosed with or treated for depression within the last year. Additional eligibility criteria were having sufficient internet literacy and German language proficiency, providing contact details, and giving online informed consent."

4a-i) Computer / Internet literacy

Reported, see 4a).

4a-ii) Open vs. closed, web-based vs. face-to-face assessments:

"The study was promoted as being on 'stress and psychological well-being' on a publicly accessible study website [26]. The aim of evaluating internet-based depression screening was not explicitly communicated, but interested participants were informed that some of them will get feedback on a part of their answers. Traditional and social media campaigns as well as print advertisements in public areas of several German cities were used to approach interested individuals. To reach a sample that strives for representativeness of the German population with respect to age and gender, a marketing company further advertised the study via a nationwide internet-based access survey panel. "

"Double entries identified based on personal data by a privacy-preserving record linkage service [27] were automatically re-allocated to their former study arm. Research staff were masked to allocation at any time until breaking the blind."

"Web-based follow-up assessments were set at one month and six months after randomisation. Two to five days and six months after randomisation, participants were contacted via telephone for complementary diagnostic interviews."

4a-iii) Information giving during recruitment

"The study was promoted as being on ‘stress and psychological well-being’ on a publicly accessible study website [26]. The aim of evaluating internet-based depression screening was not explicitly communicated, but interested participants were informed that some of them will get feedback on a part of their answers."

4b) CONSORT: Settings and locations where the data were collected

"The study was promoted as being on ‘stress and psychological well-being’ on a publicly accessible study website [26].”

“Traditional and social media campaigns as well as print advertisements in public areas of several German cities were used to approach interested individuals across Germany.”

4b-i) Report if outcomes were (self-)assessed through online questionnaires

"Web-based follow-up assessments were set at one month and six months after randomisation, with up to ten automatic email reminders being sent to participants in case of incomplete surveys. "

4b-ii) Report how institutional affiliations are displayed

Figure 1 shows how the logos of the University Medical Center Hamburg and of the German Research Foundation were displayed to participants.

5) CONSORT: Describe the interventions for each group with sufficient details to allow replication, including how and when they were actually administered

5-i) Mention names, credential, affiliations of the developers, sponsors, and owners

The software was developed by the authors together with an IT specialist. The software is not commercially available.

5-ii) Describe the history/development process

"The feedback was developed in a multistage process involving patient representatives [33, 34], an IT specialist, and a digital graphic agency to adapt the material to the possibilities of internet-based presentation."

5-iii) Revisions and updating

The intervention was not revised during the trial and only this original version was deployed.

5-iv) Quality assurance methods

“Double entries identified based on personal data by a privacy-preserving record linkage service [27] were automatically re-allocated to their former study arm.”

5-v) Ensure replicability by publishing the source code, and/or providing screenshots/screen-capture video, and/or providing flowcharts of the

"Participants of the feedback arms received information on follow-up procedures and were offered feedback on their screening result by clicking on a ‘next’-button (Figure 1)."

"Illustrations of the complete nontailored and tailored feedback versions can be found in supplements B and C."

5-vi) Digital preservation

The feedback is accessible (www.discover-studie.de/rueckmeldung; www.discover-studie.de/personalisierte-rueckmeldung); the intervention is however not archived.

5-vii) Access

"The study was promoted as being on 'stress and psychological well-being' on a publicly accessible study website [26]. "

5-viii) Mode of delivery, features/functionalities/components of the intervention and comparator, and the theoretical framework

"After completing the baseline survey, all participants were thanked for participating in the study. Participants of the feedback arms received information on follow-up procedures and were offered feedback on their screening result by clicking on a 'next'-button (Figure 1). Both nontailored and tailored feedback comprised four sections: (1) the depression screening result, (2) a note to seek diagnostic consultation by a health professional together with a link to make an appointment within the next two weeks, (3) brief general information on depression, and (4) information on depression treatment based on the German National Clinical Practice Guideline for Unipolar Depression [32]. Notably, in the German health care system depression care is available and covered by the social health insurance. Information was extended by direct links to referenced health or social services (e.g. web-based therapies covered by the health insurance, self-help groups), and the feedback form could be downloaded in a file that included all active links. In extension to the nontailored feedback, the information in the tailored feedback intervention was personalised to participants' characteristics (e.g., 'You have indicated that you had low spirits, sleep disturbances, and loss of energy during the past two weeks.'). Additionally, after being provided with the screening result (section 1) but before receiving further information (sections 2 to 4), participants were asked whether they think that their symptoms were indications of depression and whether they worried about the symptoms. According to the participants' answers, the following three feedback sections were arranged in a differing order, phrased slightly differently, and extended by information tailored to participants' risk profile (e.g. 'Depression in pregnancy is common.'). The feedback was developed in a multistage process involving patient representatives [33, 34] and an IT specialist and a digital graphic agency to adapt the material to the possibilities of web-based presentation. Illustrations of the complete nontailored and tailored feedback versions can be found in Multimedia Appendices 3 and 4."

5-ix) Describe use parameters

This intervention is a one-time use intervention.

5-x) Clarify the level of human involvement

There was no human involvement in the feedback interventions. There was only huan involvement in the telephone assessments, as already mentioned.

5-xi) Report any prompts/reminders used

"Internet-based follow-up assessments were set at one month and six months after randomisation, with up to ten automatic email reminders being sent to participants in case of incomplete surveys. Two to five days and six months after randomisation, participants were contacted via telephone for complementary diagnostic interviews, with calls being repeated at different hours during daytime and evening in case participants were not reached (see [16] for more detailed information on the data collection). "

5-xii) Describe any co-interventions (incl. training/support)

There are no co-interventions in this trial.

6a) CONSORT: Completely defined pre-specified primary and secondary outcome measures, including how and when they were assessed

Measures

“Depression diagnosis by a health professional was assessed at six months with the question: “Have you been diagnosed with depression or burnout in the last six months?”.Guideline-based depression treatment, i.e. psychotherapy and/or pharmacotherapy with antidepressant medication recommended by the German National Clinical Practice Guideline for Unipolar Depression [32], was assessed at six months with the questions: “Have you started any psychotherapy or similar treatment in the last 6 months [which]?") and “Have you started taking medication to treat depression or other complaints such as sleep problems, anxiety or stress [which ones]?". Participants could choose from guideline-based treatment options or give open answers. In case of open answers, these were checked for guideline-conformity independently by two of the authors (SK and FS).Criteria for major depression at baseline were assessed with the depression-related modules of the Structured Clinical Interviews for DSM-5 Disorders (SCID-5-CV) [35] two to five days after screening. The interviewers (BSc psychology students) were trained and supervised by the project leader, who is an experienced psychotherapist. Participants who did not meet the criteria for a major depression were considered false positive screens.Depression severity was assessed with the PHQ-9 at one and six months after screening. In accordance with the DSM-5 diagnostic criteria, the PHQ-9 assesses nine depressive symptoms each rated in terms of frequency during the past two weeks (0–3; not at all to nearly every day), resulting in a total score ranging from 0 to 27. The PHQ-9 is among the most frequently used self-report depression questionnaires, has good psychometric properties, and is sensitive to change [25, 36].Suicidal ideation was assessed with the PHQ-9 suicide item (item 9): “Over the last two weeks, how often have you been bothered by thoughts that you would be better off dead or of hurting yourself in some way?”, rated from 0–3 (not at all, several days, more than half the days, nearly every day). Emotional response to depressive symptoms was assessed with a composite scale based on two items of the Brief Illness Perception Questionnaire (Brief IPQ) that cover emotional representations of depressive symptoms: “How concerned are you about your symptoms?” and ”How much do your symptoms affect you emotionally? (e.g. do they make you angry, scared, upset or depressed)?”. The items were assessed directly after the PHQ-9 and were scored on a Likert scale ranging from 0 (not at all) to 10 (extremely). Item scores were pooled for the composite scale, resulting in one total scale ranging from 0 to 10. The respective items of the Brief IPQ showed good psychometric properties [37].”

Outcomes

“Participants were classified as misdiagnosed or mistreated if they reported having received a depression diagnosis by a health professional or guideline-based depression treatment while not having met the criteria for major depression at baseline (SCID), i.e. while being screened false positive. Deterioration in depression severity was defined as a pre-post change score of at least 4.4 points in the PHQ-9. The cut-off is based on the reliable change index (RCI), a psychometric criterion to evaluate whether a change in symptoms is considered statistically reliable, i.e. not attributable to measurement error [38]. The RCI was calculated using the PHQ-9 standard deviation from the current sample (SDbaseline = 4), the reliability coefficient from the PHQ-9 validation study (rtt = 0.84) [39], and a 95% confidence level. Deterioration in emotional response to depressive symptoms was defined as a pre-post change score of at least 3.1 points in the relating composite scale. The RCI was calculated using the standard deviation of this composite scale (SDbaseline = 1.9), the pooled reliability coefficients from the Brief IPQ validation study (rtt = 0.66), and a 95% confidence level.Deterioration in suicidal ideation was defined as the pre-post change score of at least 1 point in the PHQ-9 suicide item."

6a-i) Online questionnaires: describe if they were validated for online use and apply CHERRIES items to describe how the questionnaires were designed/deployed.

The PHQ-9 is preliminarily validated for online use: "Depression was screened as part of the baseline survey using the digitised PHQ-9 [25, 28] (see the outcomes section for further information and supplement A for the layout of the digitised version). At the standard cut-off value of ≥10 points, the paper-pencil PHQ-9 demonstrates high discriminatory performance for detecting major depression: Based on a recent individual participant data meta-analysis of studies with a semi-structured interview reference standard, pooled PHQ-9 sensitivity and specificity (95% confidence interval) were 0.85 (0.79 to 0.89) and 0.85 (0.82 to 0.87), respectively [29]. Preliminary evidence suggests that psychometric characteristics are comparable for the digitised version [30, 31]."

Other outcomes are not validated for online use.

6a-ii) Describe whether and how "use" (including intensity of use/dosage) was defined/measured/monitored

"Of the 787 participants randomised to receive any feedback, in total 744 (95%) opened the feedback screen, of which 464 (62%) downloaded the PDF and 248 (33%) interacted with the feedback by clicking at least one link or modal. There was no descriptive difference between the feedback engagement across feedback arms (see main paper, for results per study arm)."

6a-iii) Describe whether, how, and when qualitative feedback from participants was obtained

Qualitative feedback via interviews was obtained in a separate study.

6b) CONSORT: Any changes to trial outcomes after the trial commenced, with reasons

"We conducted small deviations from the preregistration: we added the outcomes misdiagnosis and emotional response to symptoms, as we deemed this of clinical interest."

7a) CONSORT: How sample size was determined

7a-i) Describe whether and how expected attrition was taken into account when calculating the sample size

Sample size calculation referred to the main analysis. It took into account a dropout rate of 35% and is described in the main paper.

7b) CONSORT: When applicable, explanation of any interim analyses and stopping guidelines

There were no interim analyses.

8a) CONSORT: Method used to generate the random allocation sequence

"After completing baseline assessment and screening, eligible participants were automatically randomised (random permuted blocks randomisation stratified for depression severity generated by a statistician) and allocated 1:1:1 to one of the three study arms. Research staff were masked to allocation at any time until breaking the blind. Due to the design, participants could not be masked but were kept unaware of trial hypotheses to minimise expectancy bias." "

8b) CONSORT: Type of randomisation; details of any restriction (such as blocking and block size)

See 8a).

9) CONSORT: Mechanism used to implement the random allocation sequence (such as sequentially numbered containers), describing any steps taken to conceal the sequence until interventions were assigned

See 8a).

10) CONSORT: Who generated the random allocation sequence, who enrolled participants, and who assigned participants to interventions

The randomization sequence was generated by a statistician, uploaded to the platform by the IT specialist, and assigned automatically in order of enrollment.

11a) CONSORT: Blinding - If done, who was blinded after assignment to interventions (for example, participants, care providers, those assessing

“Research staff were masked to allocation at any time until breaking the blind. Due to the design, participants could not be masked but were kept unaware of trial hypotheses to minimise expectancy bias."

11a-i) Specify who was blinded, and who wasn’t

“Research staff were masked to allocation at any time until breaking the blind. Due to the design, participants could not be masked but were kept unaware of trial hypotheses to minimise expectancy bias."

11a-ii) Discuss e.g., whether participants knew which intervention was the “intervention of interest” and which one was the “comparator”

"Due to the design, participants could not be masked but were kept unaware of trial hypotheses to minimise expectancy bias."

11b) CONSORT: If relevant, description of the similarity of interventions

The content of the nontailored and tailored feedback was broadly similar (see 5-viii).

12a) CONSORT: Statistical methods used to compare groups for primary and secondary outcomes

"We compared the rates of negative effects between study arms in terms of relative risks (RR). The RR estimates how much higher (or lower) the probability of negative effects is for participants in the respective feedback arm compared to the no feedback arm. To directly estimate the RR with 95% confidence intervals, we applied generalised linear models with a log link and robust sandwich variance estimator using modified log-Poisson regressions [41]. We chose this approach over alternative models as it is suited as well in case of frequent outcomes and suffers least from convergence problems [42, 43]."

12a-i) Imputation techniques to deal with attrition / missing values

“Additionally, we performed sensitivity analyses in the intention-to-treat (ITT) sample, both with and without missing data imputation. We used two strategies for imputing data: assuming that all drop-outs were deteriorators, considering this to be the most conservative estimate (worst case); and assuming that all drop-outs were non-deteriorators, considering this to be the most optimistic estimate (best case)."

12b) CONSORT: Methods for additional analyses, such as subgroup analyses and adjusted analyses

"To test for differential effects in the subgroup of false positive screened participants, we ran another series of models additionally including false-positive screens and the false-positive screen x study arm interaction term."

X26) REB/IRB Approval and Ethical Considerations [recommended as subheadingunder "Methods"] (not a CONSORT item)

"Online informed consent via checkboxes was obtained from all participants. The study was approved by the Ethics Committee of the Hamburg Medical Chamber and followed appropriate Consolidated Standards of Reporting Trials (CONSORT) guidelines, including the harms and the e-health statement (see supplement A) [9, 20-24]."

X26-iii) Safety and security procedures

"Due to ethical considerations, all participants who have indicated elevated suicidal ideation (PHQ-9 suicide item ≥ 2; more than half the days) were shown a screen providing an advice to urgently seek help and relevant information on available help services (e.g. general practitioner, local psychiatric emergency units, and the national emergency number; supplement D)."

RESULTS

13a) CONSORT: For each group, the numbers of participants who were randomly assigned, received intended treatment, and were analysed for the primary outcome

See CONSORT flow chart, Figure 2.

13b) CONSORT: For each group, losses and exclusions after randomisation, together with reasons

See CONSORT flow chart, Figure 2.

13b-i) Attrition diagram

See CONSORT flow chart, Figure 2.

14a) CONSORT: Dates defining the periods of recruitment and follow-up

"Recruitment took place from January 2021 to January 2022."

"Data collection was conducted online and in German language between January 12, 2021, and September 30, 2022."

14a-i) Indicate if critical "secular events" fell into the study period

No secular events impacted this study.

14b) CONSORT: Why the trial ended or was stopped (early)

This is not applicable to this study as it was not stopped early.

15) CONSORT: A table showing baseline demographic and clinical characteristics for each group

See Table 1 of the manuscript.

15-i) Report demographics associated with digital divide issues

See Table 1 of the manuscript.

16a) CONSORT: For each group, number of participants (denominator) included in each analysis and whether the analysis was by original

assigned groups

16-i) Report multiple "denominators" and provide definitions

See Table 2.

16-ii) Primary analysis should be intent-to-treat

"We performed this secondary analysis in the per protocol sample which included 948 (88%) out of 1078 randomised participants who had at least one post-baseline value of one of the outcomes and no major protocol violation. The latter were pre-defined as not receiving or adhering to the intervention (i.e., feedback not opened, feedback reading time less than 15 seconds or no download of feedback form), multiple participation (post-hoc data check or self-report), reports of not having answered the survey seriously, baseline survey completion time less than two minutes and provision of an invalid email address. We preferred per protocol over intention-to-treat analysis, as the second is likely to underestimate the risk of an event by inflating the denominator with participants who have provided invalid data or have never received the intervention. Whereas this is conservative in efficacy evaluations, in the current case of a risk evaluation we consider it more appropriate to prevent failing to detect a risk than overestimating it [40]. Additionally, we performed sensitivity analyses in the intention-to-treat (ITT) sample, both with and without missing data imputation. We used two strategies for imputing data: assuming that all drop-outs were deteriorators, considering this to be the most conservative estimate (worst case); and assuming that all drop-outs were non-deteriorators, considering this to be the most optimistic estimate (best case)."

17a) CONSORT: For each primary and secondary outcome, results for each group, and the estimated effect size and its precision (such as 95%

confidence interval)

"Misdiagnosis six months after screening was not associated with nontailored (RR=1.3, p=0.509) or tailored feedback (RR=1.09, p=0.843) as compared to no feedback, with rates of 4.9%, 4.1% and 3.5% in the nontailored, the tailored, and the no feedback arm, respectively. Mistreatment six months after screening was not associated with nontailored (RR=0.87, p=0.645) nor tailored feedback (RR=0.95, p=0.859), either, with rates of 7.2%, 7.7%, and 8.3% in the nontailored, the tailored, and the no feedback arm. Descriptively, the rate of mistreatment was higher for psychotherapy (4.9%, 6.4%, and 6.2%) compared to pharmacotherapy (3.4%, 2.2%, and 2.8%). Deterioration in depression severity was not associated with nontailored (one month: RR=1.96, p=0.095; six months: RR=0.6, p=0.143) or tailored feedback (one month: RR=0.7, p=0.494; six

months: RR=0.74, p=0.366), with rates of 5.7%, 2.0%, and 2.9% at one month and 4.1%, 5.1%, and 6.8% at six months in the nontailored, tailored, and no feedback study arm. Deterioration in emotional response to depressive symptoms was not associated with nontailored (one month: RR=1.18, p=0.750; six months: RR=0.46, p=0.197) or tailored feedback (one month: RR=0.23, p=0.128; six months: RR=0.7, p=0.491) either, with rates of 2.7%, 0.7%, and 2.3% at one month and 1.4%, 2%, and 2.9% at six months. Deterioration in suicidal ideation was not associated with nontailored (RR=1.12, p=0.655) or tailored feedback (RR=1.4, p=0.147) at six months, with rates of 10.5%, 13.1%, and 9.4%. At one month, it was almost two-fold increased in the nontailored (RR=1.92; p=0.014), but not in the tailored feedback arm (RR=1.26, p=0.427), as compared to no feedback. Rates in the nontailored, the tailored, and the no feedback arm were 12.3%, 8.1%, and 6.4%. Absolute numbers and rates for all negative effects per study arm and time point are shown in Table 2. Relative risks with corresponding 95% confidence intervals are illustrated in Figure 3."

17a-i) Presentation of process outcomes such as metrics of use and intensity of use

There were no process outcomes assessed.

17b) CONSORT: For binary outcomes, presentation of both absolute and relative effect sizes is recommended

See Table 2 of the manuscirpt.

18) CONSORT: Results of any other analyses performed, including subgroup analyses and adjusted analyses, distinguishing pre-specified from exploratory

"Results did not differ for the subgroup of false-positives (Pinteraction ranging between 0.287 and 0.804). Sensitivity analyses based on logistic regression models as well as those in the ITT sample with the full analysis set and with missing data imputation based on the best case scenario showed comparable results. In the ITT analysis based on the worst case scenario, however, the relative risk for deterioration in suicidal ideation in the nontailored feedback arm at one month was not higher than in the no feedback arm (RR=1.26, p=0.065; supplement D). Post hoc analyses exploring baseline demographic and clinical characteristics of all participants deteriorated in any outcome at any time point were comparable to the total sample (supplement F)."

18-i) Subgroup analysis of comparing only users

As mentioned, the per protocol analysis was our main analysis, as this seemd mre appropriate in the context of negative effects (see above).

19) CONSORT: All important harms or unintended effects in each group

All results relate to negative effects.

19-i) Include privacy breaches, technical problems

There were no privacy breaches or unexpected/unintended incidents.

19-ii) Include qualitative feedback from participants or observations from staff/researchers

No qualitative feedback was collected in this study.

DISCUSSION

20) CONSORT: Trial limitations, addressing sources of potential bias, imprecision, multiplicity of analyses

20-i) Typical limitations in ehealth trials

"The interpretation of these results should be considered in the context of the study's limitations. First, the underlying DISCOVER trial did not explicitly call for those seeking depression screening. As these may be more eager to follow the advice of the feedback, in this sample misdiagnosis and mistreatment might be underestimated. A second limitation is that due to the design of the DISCOVER trial, the selection of outcomes was limited and relevant negative effects such as distress, stigma, treatment side effects, or overdiagnosis (i.e. the diagnosis of correctly diagnosed but mild cases that would not benefit from treatment [14]) could not be assessed. Third, all outcomes were self-reported. Although this is common in psychological interventions, particularly the assessment of misdiagnosis and mistreatment would benefit from more objetive data from

health care providers. Fourth, the operationalisations of suicidal ideation and emotional response to depressive symptoms are based on a single item and a composite score not well validated for this purpose. Indeed, evidence for the validity of the PHQ-9 suicide item is inconclusive, with studies indicating both good prediction versus overestimation of suicidal ideation or attempts [44, 45]. Lastly, the study was planned post-hoc and therefore not powered to detect the selected outcomes, and multiple testing might have led to overestimation of significance in the case of deterioration in suicidal ideation. Notably, the findings refer to the German health care system where psychotherapy is available and covered by the social health insurance. Particularly rates for misdiagnosis

21) CONSORT: Generalisability (external validity, applicability) of the trial findings

21-i) Generalizability to other populations

See 20-i).

21-ii) Discuss if there were elements in the RCT that would be different in a routine application setting

This intervention was delivered as design for practice.

22) CONSORT: Interpretation consistent with results, balancing benefits and harms, and considering other relevant evidence

22-i) Restate study questions and summarize the answers suggested by the data, starting with primary outcomes and process outcomes (use)

"To the best of our knowledge, this secondary analysis is the first study to systematically examine potential negative effects of feedback after internet-based depression screening in a large sample of currently undiagnosed and untreated individuals with at least moderate depression severity. The results indicate that feedback, both nontailored and tailored, was not associated with increased rates of misdiagnosis, mistreatment, deterioration in depressive symptoms, or deterioration in emotional response to symptoms as compared to no feedback. Deterioration of suicidal ideation, however, appeared to be more likely one month after receiving nontailored feedback compared with no feedback; an association that was not found any more at six-months follow-up and neither after tailored feedback. Although almost 40% of the sample turned out to be screened false positive, irrespective of the study arm rates of subsequent misdiagnosis and mistreatment were lower than 5% and 9%, respectively, with rates of pharmacotherapy ranging even lower than 4%. Across study arms, deterioration in emotional response to depressive symptoms was reported by at most 3% of participants, deterioration of depression severity by at most 7%, and deterioration of suicidal ideation by at most 13%."

22-ii) Highlight unanswered new questions, suggest future research

"Therefore, comparing outcomes such as distress or negative affectivity shortly after providing the screening vs. the feedback appears worthwile to further address these issues (see [48, 49] for examplary study designs in suicide screening)."

"Given that these results should be interpreted with caution due to the study’s limitations, more robust research is needed to further address suicidal ideation in internet-based depression screening. If prospective trials that use validated ouctome measures corroborate an association of internet-based screening and/or feedback with suicidal ideation, this should inform regulations of currently unmonitored internet-based depression tests. Further, the findings should also inform research regarding comparable depression screening in medical and primary care settings, which is currently recommended in many countries despite very uncertain evidence regarding potential harms [51]."

OTHER INFORMATION

23) CONSORT: Registration number and name of trial registry

Trial Registration: ClinicalTrials.gov (NCT04633096)
Preregistration of secondary data analysis: OSF.io (https://osf.io/tzyrd)

24) CONSORT: Where the full trial protocol can be accessed, if available

Sikorski, F., et al., The efficacy of automated feedback after internet-based depression screening: Study protocol of the German, three-armed, randomised controlled trial DISCOVER. Internet Interventions, 2021. 25: p. 100435.

25) CONSORT: Sources of funding and other support (such as supply of drugs), role of funders

"This work was funded by the German Research Foundation as part of the underlying DISCOVER RCT (grant number: 424162019)."

X27-i) State the relation of the study team towards the system being evaluated

In terms of conflicts, "None declared".
