# Supplementary material for: Potential Harms of Feedback After Web-Based Depression Screening: Secondary Analysis of Negative Effects in the Randomized Controlled DISCOVER Trial
Source: J Med Internet Res. 2025 Apr 30;27:e59476. doi: 10.2196/59476 (PMC12079080; doi:10.2196/59476)
Supplement: Multimedia Appendix 6 [file jmir_v27i1e59476_app6.docx]

**Multimedia Appendix: Characteristics of ITT sample**

Table. Baseline demographic and clinical characteristics of the intention-to-treat sample (N=1178).

|  |  | No feedback (n=391) | Non-tailored feedback (n=393) | Tailored feedback (n=394) |
| --- | --- | --- | --- | --- |
| **Age, years** | | 36.5 (13.8) | 37.7 (14.0) | 37.2 (14.8) |
| **Gender** | |  |  |  |
|  | Female | 276 (71%) | 275 (70%) | 273 (69%) |
|  | Male | 111 (28%) | 115 (30%) | 118 (30%) |
|  | Diverse | 4 (1%) | 3 (1%) | 3 (1%) |
| **German mother tongue** | | 369 (94%) | 370 (94%) | 379 (96%) |
| **Migration background** | | 47 (12%) | 35 (9%) | 39 (10%) |
| **Being in a relationship** | | 167 (43%) | 200 (51%) | 192 (49%) |
| **Living together** | | 258 (66%) | 277 (71%) | 265 (67%) |
| **Formal school education** | |  |  |  |
|  | Low (less than 10 years) | 71 (18%) | 80 (20%) | 68 (17%) |
|  | Middle (at least 10 years) | 120 (31%) | 129 (33%) | 134 (34%) |
|  | High (A-level or above) | 200 (51%) | 184 (47%) | 192 (49%) |
| **Working** | | 276 (71%) | 278 (71%) | 293 (74%) |
| **Quality of life (EQ-5D-5L VAS)** | | 57.7 (22.4) | 56.8 (22.2) | 58.6 (22.0) |
| **Depression severity (PHQ-9)** | | 14.8 (4.0) | 14.8 (4.1) | 14.7 (3.9) |
| **Emotional response** | | 6.9 (2.1) | 6.9 (2) | 7 (1.8) |
| **Anxiety severity (GAD-7)** | | 12.0 (4.3) | 12.3 (4.3) | 11.9 (4.3) |
| **Somatic symptom severity (SSS-8)** | | 14.5 (5.3) | 14.5 (5.1) | 14.4 (5.3) |
| **No. of depression-related risk factors^a^** | | 6.0 (2.5) | 6.1 (2.4) | 5.8 (2.3) |
| **Frequency of suicidal ideation within last two weeks** | |  |  |  |
|  | None | 167 (51%) | 161 (51%) | 165 (54%) |
|  | Several days | 98 (30%) | 113 (36%) | 94 (31%) |
|  | More than half the days | 37 (11%) | 23 (7%) | 26 (9%) |
|  | Nearly every day | 25 (8%) | 17 (5%) | 22 (7%) |
| **Self-identifying as suffering from depression** | |  |  |  |
|  | No | 55 (14%) | 44 (11%) | 66 (17%) |
|  | Maybe | 162 (41%) | 201 (51%) | 176 (45%) |
|  | Yes | 174 (45%) | 148 (38%) | 152 (39%) |
| **Meeting criteria for major depression** **(SCID)** | | 194 (62%)^b^ | 180 (61%)^c^ | 180 (60%)^d^ |

Data are mean (SD) or n (%). PHQ-9=Patient Health Questionnaire-9. EQ-5D-5L=EuroQoL-5 Dimensions-5 Level scale. VAS=visual analogue scale. GAD-7=Generalized Anxiety Disorder-7. SSS-8=Somatic Symptom Scale. ^a^Risk factors included self-reported anxiety, addiction, traumatic life events, persistent physical symptoms, mood swings, chronic physical condition, lack of social support, mental comorbidity, mental comorbidity in family, history of suicide, current pregnancy, post-natal phase, menopause, premenstrual syndrome. SCID=Structured Clinical Interview for DSM-5 Disorders; the interview was conducted approximately 2 to 5 days after randomisation. ^b^78 cases with missing data. ^c^97 cases with missing data. ^d^94 cases with missing data.
