# Supplementary material for: Potential Harms of Feedback After Web-Based Depression Screening: Secondary Analysis of Negative Effects in the Randomized Controlled DISCOVER Trial
Source: J Med Internet Res. 2025 Apr 30;27:e59476. doi: 10.2196/59476 (PMC12079080; doi:10.2196/59476)
Supplement: Multimedia Appendix 7 [file jmir_v27i1e59476_app7.docx]

**Multimedia Appendix: Sensitivity analyses**

**Table 7.1**. Rates and relative risks of negative effects per study arm and time point in the intention-to-treat sample in full analysis set.

|  |  |  |  |  |  |  |  | Relative risk (95% CI) | |
| --- | --- | --- | --- | --- | --- | --- | --- | --- | --- |
|  |  | N | NF | N | NTF | N | TF | NTF vs. NF | TF vs. NF |
| **Misdiagnosis** | | 313^a^ | 11 (3.5%) | 296 ^a^ | 13 (4.4%) | 300 ^a^ | 11 (3.7%) | 1.25 [0.57 – 2.75] | 1.04 [0.46 – 2.37] |
| **Mistreatment** | | 313 ^a^ | 24 (7.7%) | 296 ^a^ | 19 (6.4%) | 300 ^a^ | 21 (7%) | 0.84 [0.47 – 1.5] | 0.91 [0.52 – 1.6] |
|  | **PSYCHOTHERAPY** | 313 ^a^ | 18 (5.8%) | 296 ^a^ | 13 (4.4%) | 300 ^a^ | 17 (5.7%) | 0.76 [0.38 – 1.53] | 0.99 [0.52 – 1.88] |
|  | **PHARMACOTHERAPY** | 313 ^a^ | 8 (2.6%) | 296 ^a^ | 9 (3%) | 300 ^a^ | 6 (2%) | 1.19 [0.47 – 3.04] | 0.78 [0.28 – 2.23] |
| **Deterioration in depression** | |  |  |  |  |  |  |  |  |
|  | **1 month** | 329 | 11 (3.3%) | 325 | 18 (5.5%) | 322 | 7 (2.2%) | 1.66 [0.8 – 3.45] | 0.65 [0.26 – 1.66] |
|  | **6 months** | 325 | 22 (6.8%) | 319 | 14 (4.4%) | 321 | 18 (5.6%) | 0.65 [0,34 – 1.24] | 0,83 [0.45 – 1.52] |
| **Deterioration in suicidality** | |  |  |  |  |  |  |  |  |
|  | **1 month** | 329 | 25 (7.6%) | 325 | 42 (12.9%) | 322 | 28 (8.7%) | 1.7 [1.06 – 2.72] | 1.14 [0.68 – 1.92] |
|  | **6 months** | 325 | 33 (10.2%) | 319 | 34 (10.7%) | 321 | 44 (13.7%) | 1.05 [0.67 – 1.65] | 1.35 [0.88 – 2.06] |
| **Deterioration in emotional response** | |  |  |  |  |  |  |  |  |
|  | **1 month** | 325 | 9 (2.8%) | 323 | 9 (2.8%) | 320 | 3 (0.9%) | 1.06 [0.41 – 2.76] | 0.32 [0.07 – 1.48] |
|  | **6 months** | 322 | 9 (2.8%) | 317 | 6 (1.9%) | 317 | 8 (2.5%) | .44 [0.14 – 1.37] | 0.76 [0.29 – 2] |

Data are n (%). NF=No feedback arm. NTF=Nontailored feedback arm. TF=Tailored feedback arm. CI=Confidence Interval.

**Table 7.2.** Rates and relative risks of negative effects per study arm and time point in the intention-to-treat sample with missing data imputation; missing data = 0 – best case.

|  |  |  |  |  |  |  |  | Relative risk (95% CI) | |
| --- | --- | --- | --- | --- | --- | --- | --- | --- | --- |
|  |  | N | NF | N | NTF | N | TF | NTF vs. NF | TF vs. NF |
| **Misdiagnosis** | | 391 | 11 (2.8%) | 393 | 13 (3.3%) | 394 | 11 (2.8%) | 1.18 [0.53 – 2.59] | 0.99 [0.44 – 2.26] |
| **Mistreatment** | | 391 | 24 (6.1%) | 393 | 19 (4.8%) | 394 | 21 (5.3%) | 0.79 [0.44 – 1.41] | 0.87 [0.49 – 1.53] |
|  | **PSYCHOTHERAPY** | 391 | 18 (4.6%) | 393 | 13 (3.3%) | 394 | 17 (4.3%) | 0.72 [0.36 – 1.45] | 0.94 [0.49 – 1.79] |
|  | **PHARMACOTHERAPY** | 391 | 8 (2%) | 393 | 9 (2.3%) | 394 | 6 (1.5%) | 1.12 [0.44 – 2.87] | 0.74 [0.26 – 2.13] |
| **Deterioration in depression** | |  |  |  |  |  |  |  |  |
|  | **1 month** | 391 | 11 (2.8%) | 393 | 18 (4.6%) | 394 | 7 (1.8%) | 1.63 [0.78 – 3.4] | 0.63 [0.25 – 1.61] |
|  | **6 months** | 391 | 22 (5.6%) | 393 | 14 (3.6%) | 394 | 18 (4.6%) | 0.63 [0.33 – 1.22] | 0.81 [0.44 – 1.5] |
| **Deterioration in suicidality** | |  |  |  |  |  |  |  |  |
|  | **1 month** | 391 | 25 (6.4%) | 393 | 42 (10.7%) | 394 | 28 (7.1%) | 1.67 [1.04 – 2.67] | 1.11 [0.66 – 1.87] |
|  | **6 months** | 391 | 33 (8.4%) | 393 | 34 (8.7%) | 394 | 44 (11.2%) | 1.03 [0.65 – 1.62] | 1.32 [0.86 – 2.03] |
| **Deterioration in emotional response** | |  |  |  |  |  |  |  |  |
|  | **1 month** | 391 | 9 (2.3%) | 393 | 9 (2.3%) | 394 | 3 (0.8%) | 1 [0.4 – 2.48] | 0.33 [0.9 – 1.21] |
|  | **6 months** | 391 | 9 (2.3%) | 393 | 6 (1.5%) | 394 | 8 (2%) | 0.66 [0.24 – 1.85] | 0.88 [0.34 – 2.26] |

Data are n (%). NF=No feedback arm. NTF=Nontailored feedback arm. TF=Tailored feedback arm. CI=Confidence Interval.

**Table 7.3**. Rates and relative risks of negative effects per study arm and time point in intention-to-treat sample with missing data imputation; missing data = 1 – worst case.

|  |  |  |  |  |  |  |  | Relative risk (95% CI) | |
| --- | --- | --- | --- | --- | --- | --- | --- | --- | --- |
|  |  | N | NF | N | NTF | N | TF | NTF vs. NF | TF vs. NF |
| **Misdiagnosis** | | 391 | 98 (25.1%) | 393 | 116 (29.5%) | 394 | 112 (28.4%) | 1.18 [0.94 – 1.48] | 1.13 [0.9 – 1.43] |
| **Mistreatment** | | 391 | 102 (26%) | 393 | 116 (29.5%) | 394 | 116 (29.4%) | 1.13 [0.9 – 1.42] | 1.13 [0.9 – 1.42] |
|  | **PSYCHOTHERAPY** | 391 | 96 (24.6%) | 393 | 110 (28%) | 394 | 111 (28.2%) | 1.14 [0.9 -1.44] | 1.15 [0.91 – 1.45] |
|  | **PHARMACOTHERAPY** | 391 | 86 (22%) | 393 | 106 (27%) | 394 | 100 (25.4%) | 1.23 [0.96 – 1.57] | 1.15 [0.9 – 1.49] |
| **Deterioration in depression** | |  |  |  |  |  |  |  |  |
|  | **1 month** | 391 | 73 (18.7%) | 393 | 86 (21.9%) | 394 | 79 (20.1%) | 1.17 [0.89 – 1.55] | 1.07 [0.81 – 1.43] |
|  | **6 months** | 391 | 88 (22.5%) | 393 | 88 (22.4%) | 394 | 91 (23.1%) | 1 [0.77 – 1.29] | 1.03 [0.79 – 1.33] |
| **Deterioration in suicidality** | |  |  |  |  |  |  |  |  |
|  | **1 month** | 391 | 87 (22.3%) | 393 | 110 (28%) | 394 | 100 (25.4%) | 1.26 [0.99 – 1.61] | 1.14 [0.89 – 1.47] |
|  | **6 months** | 391 | 99 (25.3%) | 393 | 108 (27.5%) | 394 | 117 (29.7%) | 1.01 [0.86 – 1.37] | 1.17 [0.93 – 1.47] |
| **Deterioration in emotional response** | |  |  |  |  |  |  |  |  |
|  | **1 month** | 391 | 75 (19.2%) | 393 | 79 (20.1%) | 394 | 77 (19.5%) | 0.69 [0.32 – 1.52] | 0.45 [0.17 – 1.18] |
|  | **6 months** | 391 | 78 (19.9%) | 393 | 82 (20.9%) | 394 | 85 (21.6%) | 1.05 [0.79 – 1.38] | 1.08 [0.82 – 1.42] |

Data are n (%). NF=No feedback arm. NTF=Nontailored feedback arm. TF=Tailored feedback arm. CI=Confidence Interval. ^a^n refers to participants who completed both the follow-up assessment and the SCID depression module at baseline.

**Table 7.4.** Rates and odds ratios of negative effects per study arm and time point in per protocol sample, based on logistic regression.

|  |  |  |  |  |  |  |  | Odds ratios (95% CI) | |
| --- | --- | --- | --- | --- | --- | --- | --- | --- | --- |
|  |  | N | NF | N | NTF | N | TF | NTF vs. NF | TF vs. NF |
| **Misdiagnosis** | | 290^a^ | 11 (3.5%) | 263^a^ | 13 (4.9%) | 267 ^a^ | 11 (4.1.%) | 1.31 [0.58 – 3] | 1.09 [0.47 – 2.56] |
| **Mistreatment** | | 290^a^ | 24 (8.3%) | 263^a^ | 19 (7.2%) | 267 ^a^ | 21 (7.7%) | 0.86 [0.46 – 1.62] | 0.95 [0.51 - 1.74] |
|  | **Psychotherapy** | 290^a^ | 18 (6.2%) | 263^a^ | 13 (4.9%) | 267 ^a^ | 17 (6.4%) | 0.79 [0.38 – 1.64] | 1.03 [0.52 – 2.04] |
|  | **Pharmacotherapy** | 290^a^ | 8 (2.8%) | 263^a^ | 9 (3.4%) | 267 ^a^ | 6 (2.2%) | 1.25 [0.48 – 3.27] | 0.81 [0.28 – 2.37] |
| **Deterioration in depression** | |  |  |  |  |  |  |  |  |
|  | **1 month** | 312 | 9 (2.9%) | 300 | 17 (5.7%) | 297 | 6 (2.0%) | 2.02 [0.89 – 4.61] | 0.69 [0.24 – 1.98] |
|  | **6 months** | 309 | 21 (6.8%) | 296 | 12 (4.1%) | 297 | 15 (5.1%) | 0.58 [0,28 – 1.2] | 0,73 [0.37 – 1.44] |
| **Deterioration in suicidality** | |  |  |  |  |  |  |  |  |
|  | **1 month** | 312 | 20 (6.4 %) | 300 | 37 (12.3%) | 297 | 24 (8.1%) | 2.05 [1.16 – 3.63]** | 1.28 [0.69 – 2.38] |
|  | **6 months** | 309 | 29 (9.4%) | 296 | 31 (10.5%) | 297 | 39 (13.1%) | 1.13 [0.66 – 1.9] | 1.46 [0.88 – 2.43] |
| **Deterioration in emotional response** | |  |  |  |  |  |  |  |  |
|  | **1 month** | 308 | 7 (2.3%) | 299 | 8 (2.7%) | 296 | 2 (0.7%) | 1.18 [0.42 – 3.3] | 0.29 [0.06 – 1.42] |
|  | **6 months** | 307 | 9 (2.9%) | 294 | 4 (1.4%) | 299 | 6 (2%) | 0.46 [0.14 – 1.5] | 0.69 [0.24 – 1.97] |

Data are n (%). NF=No feedback arm. NTF=Nontailored feedback arm. TF=Tailored feedback arm. CI=Confidence Interval. ^a^n refers to participants who completed both the follow-up assessment and the SCID depression module at baseline.
