# Supplementary material for: Potential Harms of Feedback After Web-Based Depression Screening: Secondary Analysis of Negative Effects in the Randomized Controlled DISCOVER Trial
Source: J Med Internet Res. 2025 Apr 30;27:e59476. doi: 10.2196/59476 (PMC12079080; doi:10.2196/59476)
Supplement: Multimedia Appendix 8 [file jmir_v27i1e59476_app8.docx]

**Multimedia Appendix: Post hoc analyses**

Table. Baseline and clinical characteristics of deteriorators (in any outcome) in the per protocol sample (N=203).

| **Age, years** | | 37.4 (14.6) |
| --- | --- | --- |
| **Gender** | |  |
|  | Female | 138 (68%) |
|  | Male | 63 (31%) |
|  | Diverse | 2 (1%) |
| **German mother tongue** | | 197 (97%) |
| **Migration background** | | 17 (9%) |
| **Being in a relationship** | | 104 (51%) |
| **Living together** | | 139 (69%) |
| **Formal school education** | |  |
|  | Low (less than 10 years) | 44 (22%) |
|  | Middle (at least 10 years) | 67 (33%) |
|  | High (A-level or above) | 92 (45%) |
| **Working** | | 131 (65%) |
| **Quality of life (EQ-5D-5L VAS)** | | 56.2 (23.7) |
| **Depression severity (PHQ-9)** | | 14.3 (3.6) |
| **Emotional response to depressive symptoms (composite scale)** | | 6.8 (2) |
| **Anxiety severity (GAD-7)** | | 12 (4.5) |
| **Somatic symptom severity (SSS-8)** | | 14.5 (5.2) |
| **No. of depression-related risk factors^a^** | | 6.2 (2.4) |
| **Frequency of suicidal ideation within last two weeks (PHQ-9 item 9)** | |  |
|  | None | 125 (62%) |
|  | Several days | 61 (30%) |
|  | More than half the days | 16 (8%) |
|  | Nearly every day | 1 (0.5%) |
| **Self-identifying as suffering from depression** | |  |
|  | No | 23 (11%) |
|  | Maybe | 98 (48%) |
|  | Yes | 82 (40%) |
| **Meeting criteria for major depression (SCID)** | | 115 (70%)^b^ |

Data are mean (SD) or n (%). PHQ-9=Patient Health Questionnaire-9. EQ-5D-5L=EuroQoL-5 Dimensions-5 Level scale. VAS=visual analogue scale. GAD-7=Generalized Anxiety Disorder-7. SSS-8=Somatic Symptom Scale. ^a^Risk factors included self-reported anxiety, addiction, traumatic life events, persistent physical symptoms, mood swings, chronic physical condition, lack of social support, mental comorbidity, mental comorbidity in family, history of suicide, current pregnancy, post-natal phase, menopause, premenstrual syndrome. SCID=Structured Clinical Interview for DSM-5 Disorders; the interview was conducted approximately 2 to 5 days after randomisation. ^b^38 cases with missing data.
